# Consonantal F_0_ perturbation in American English involves multiple mechanisms

**DOI:** 10.1121/10.0004239

**Published:** 2021-04-29

**Authors:** Yi Xu, Anqi Xu

**Affiliations:** Department of Speech, Hearing and Phonetic Sciences, University College London, London, United Kingdom

## Abstract

In this study, we revisit consonantal perturbation of *F*_0_ in English, taking into particular consideration the effect of alignment of *F*_0_ contours to segments and the *F*_0_ extraction method in the acoustic analysis. We recorded words differing in consonant voicing, manner of articulation, and position in syllable, spoken by native speakers of American English in both statements and questions. In the analysis, we compared methods of *F*_0_ alignment and found that the highest *F*_0_ consistency occurred when *F*_0_ contours were time-normalized to the entire syllable. Applying this method, along with using syllables with nasal consonants as the baseline and a fine-detailed *F*_0_ extraction procedure, we identified three distinct consonantal effects: a large but brief (10–40 ms) *F*_0_ raising at voice onset regardless of consonant voicing, a smaller but longer-lasting *F*_0_ raising effect by voiceless consonants throughout a large proportion of the following vowels, and a small lowering effect of around 6 Hz by voiced consonants, which was not found in previous studies. Additionally, a brief anticipatory effect was observed before a coda consonant. These effects are imposed on a continuously changing *F*_0_ curve that is either rising-falling or falling-rising, depending on whether the carrier sentence is a statement or a question.

## I. INTRODUCTION

When a non-sonorant consonant occurs in a speech utterance, the vibration of the vocal folds is affected in two major ways. First, voicing may be interrupted, resulting in a break of otherwise continuous fundamental frequency (*F*_0_) trajectory. This can be referred to as a *horizontal disruption* or *voice break*. Second, *F*_0_ around the voice break may be raised or lowered because of the consonant. This is usually known as consonantal perturbation of *F*_0_ (Hombert *et al.*, 1979; Ohala, 1974). Other names include pitch skip (Haggard *et al.*, 1970; Hanson, 2009), micro *F*_0_ (Kohler, 1990), and CF0 (Kingston, 2007; Kirby and Ladd, 2016). We will refer to the raising and lowering effects as *vertical perturbation* in order to distinguish them from the effects of voice break. This distinction is necessary because research on the effects of consonants on *F*_0_ over the past decades has focused predominantly on vertical perturbation, while the effects of voice break have received much less attention. As will be demonstrated, the assessment and interpretation of vertical perturbation is contingent on the treatment of voice break in *F*_0_ measurement. In particular, full consideration of voice break may help answer four critical questions: (a) Are there both raising of *F*_0_ by voiceless consonants and lowering of *F*_0_ by voiced consonants? (b) Are there multiple mechanisms that jointly contribute to *F*_0_ perturbation? (c) Are there both carryover and anticipatory *F*_0_ perturbations? And (d) is *F*_0_ perturbation affected by intonation?

### A. Vertical perturbation and macro vs micro *F*_0_

**FIG. 1.**
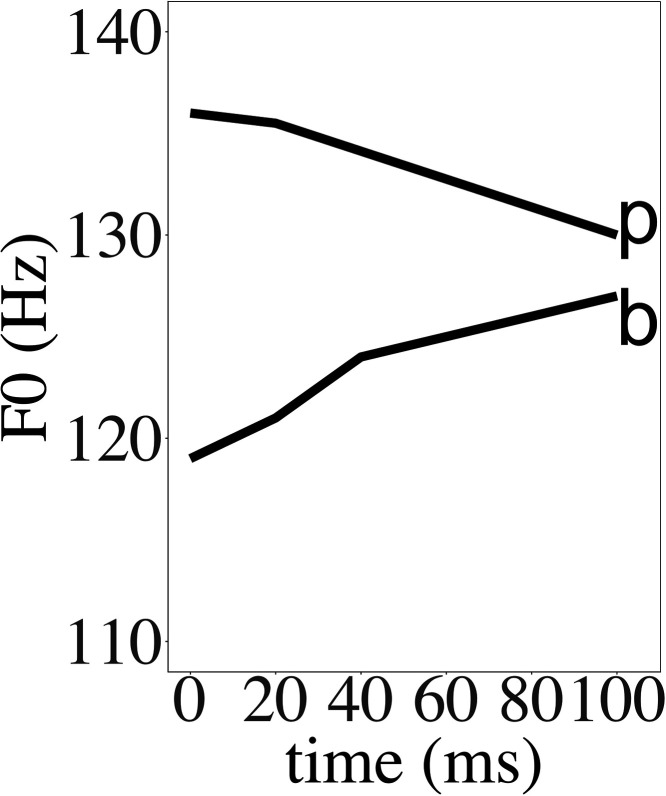
Average *F*_0_ values of vowels following English voiced and voiceless bilabial stops in real time, aligned at vowel onset (adapted from Fig. 1 in Hombert *et al.*, 1979).

As early as in the middle of the last century, House and Fairbanks (1953) measured mean *F*_0_ averaged across the entire vowel in English and found that it was higher after voiceless consonants than after voiced consonants.^1^ A similar finding was made by Lehiste and Peterson (1961) with peak *F*_0_ as the measurement. Lea (1973) investigated the time course of the consonant perturbation and found that *F*_0_ first rose after a voiceless consonant and then decreased throughout the vowel, while the opposite was true of voiced consonants. Hombert (1978) and Hombert *et al.* (1979) also reported a rise-fall dichotomy in the mean *F*_0_ curves, as shown in Fig. 1, which has since been often cited as the prototypical dichotic consonantal perturbation of *F*_0_. Later studies, however, started to show a more complex picture. Ohde (1984) and Silverman (1984) reported that *F*_0_ fell after all obstruent consonants regardless of their voicing. Hanson (2009) applied an improved method to examine the time course of *F*_0_ perturbation by including nasal consonants as the baseline. She found that *F*_0_ was raised after voiceless consonants but not lowered after voiced ones. However, the rise-fall dichotomy remains a widely accepted notion, especially in its use as a key trigger for tonogenesis (Chen *et al.*, 2017; Evans *et al.*, 2018**;** Gao and Arai, 2019; Hill, 2019).

There has been less work on the anticipatory *F*_0_ perturbation by consonants. Hombert *et al.* (1979) found no perturbation effect on the preceding vowels and Lehiste and Peterson (1961) reported that there was no consistent effect for English. Kohler (1982), however, found that *F*_0_ was lowered before voiced stops in contrast with voiceless stops when the sentence intonation is falling but not in sentences with either monotone or rising intonation. Silverman (1984) also reported a dichotomy in the preceding vowels according to consonant voicing.

As summarized above, there is still no clear consensus on vertical perturbation either as a carryover or anticipatory effect. In fact, two major issues remain unresolved. The first is the underlying cause of vertical perturbation. Two mechanisms have been proposed. The first is the aerodynamic hypothesis (Ladefoged, 1967), according to which the release of a voiceless stop is accompanied by a high rate of airflow across the glottis, which would increase the rate of vocal fold vibration. During a voiced consonant, on the other hand, the flow of air across the glottis is reduced, thus lowering pitch. The chief argument against this view is that the observed perturbatory effect lasts too long to be due to an aerodynamic effect. Löfqvist *et al.* (1995) have shown that the release of voiceless consonants is indeed accompanied by increased airflow, but only for a brief period of time, whereas vertical *F*_0_ perturbation can last for at least 100 ms (Hombert *et al.*, 1979).

An alternative hypothesis is that there is an adjustment of the tension of the vocal folds during the production of the consonant depending on voicing (Halle and Stevens, 1971). This is supported by electromyography (EMG) recordings that show higher cricothyroid (CT) activity during voiceless consonants than during voiced consonants (Dixit, 1975; Löfqvist *et al.*, 1989). Also, significant voicing differences have been found in the vertical position of the larynx (Ewan and Krones, 1974) and the pharyngeal cavity (Bell-Berti, 1975; Westbury, 1983). The changes in the tension of the vocal folds would affect phonation threshold (Berry *et al.*, 1996). In addition, the changes in laryngeal height would affect transglottal pressure (Hanson and Stevens, 2002). Both types of changes would help to stop voicing for voiceless consonants and sustain voicing for voiced consonants, but both of them would also affect *F*_0_. The problem with this hypothesis is in fact part of the second unresolved issue about vertical perturbation: do voiced consonants actually lower *F*_0_ or do they have no effects on *F*_0_? So far there is no clear evidence that *F*_0_ is lowered after voiced obstruents due to vocal folds slackening or larynx lowering. Hanson (2009) finds that *F*_0_ following phonologically voiced stops in English is actually slightly higher than the nasal baseline. Kirby and Ladd (2016) reported that even for French and Italian voiced consonants (which are phonetically prevoiced consonants), there was only a marginal *F*_0_ lowering after the oral closure according to the mean *F*_0_ contours, and the effect was not statistically significant. These results have been further replicated in Kirby *et al.* (2020).

The above two possibilities have been considered as the only two alternative mechanisms so far. There is a third possibility that has not been contemplated before, however. That is, it is also possible that an aerodynamic effect and the effect of vocal fold tension both occur, but they differ in temporal scale. The aerodynamic effect may occur right after voice onset, but fade away quickly (Löfqvist *et al.*, 1995), while the vocal fold tension effect may have a slow onset, but last longer (Hanson, 2009).

One of the reasons for the lack of consensus is that the observation of vertical perturbation may be affected by the method of its assessment. Silverman (1986) points out that the effect of consonantal perturbation cannot be properly understood unless the underlying intonation is well controlled. For example, if a consonant happens to occur in the course of a rising intonation, the *F*_0_ rise after the consonant release may not be entirely due to the consonant. He further reports that, once the underlying intonation is taken into consideration, there is no more rise-fall dichotomy due to stop voicing in English because *F*_0_ falls after both voiced and voiced stops, except that the fall in the former is shallower than in the latter. Silverman's argument is shadowed by the notion of macro versus micro F_0_ (Kohler, 1982, 1990), the first of which refers to stress and intonation, and the second to segmental effects. Kohler (1982) reported that in German the *F*_0_ divergence after voiced and voiceless consonants was large in rising or monotone contours but not in falling contours, while the effect of voicing of a following stop in *F*_0_ was observable only in falling contours.

It is not always obvious what an underlying intonation looks like around a consonant, however. Although one could infer it from the *F*_0_ trajectories before and after the consonant, it is also possible that a sharp pitch turn takes place right before, after, or even during the consonant. When that happens, the assessment of vertical perturbation becomes tricky. What is needed is a careful consideration of the relation between underlying intonation and voice break.

### B. Voice break and *F*_0_-syllable alignment

**FIG. 2.**
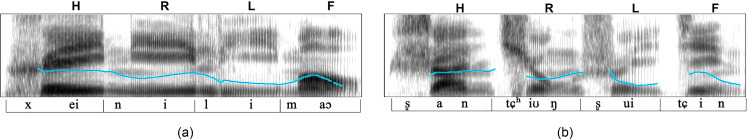
(Color online) (a) Spectrogram of utterances consisting of only vowels and sonorants; (b) spectrogram of utterances consisting of vowels and consonants.

In a sentence consisting of only vowels and sonorant consonants, like the Mandarin phrase /hei1 ni2 li3 mao4/ (black woolen hat) in Fig. 2(a) (where the numbers indicate the high, rising, low, and falling tones, respectively), the *F*_0_ trajectory would be largely smooth and continuous throughout the utterance. This is because the tension of the vocal folds, which is mainly responsible for *F*_0_, cannot change instantaneously. A voluntary pitch change of just one semitone would take over 100 ms to complete on average (Xu and Sun, 2002). Once obstruent consonants occur in an utterance, continuous *F*_0_ is interrupted by the voice breaks during the constriction and sometimes also during the release, as is the case with the Mandarin expression /shan1 qiong2 shui3 jin4/ (no way out) in Fig. 2(b). A question then arises as to whether the voice break also interrupts the continuous adjustment of vocal fold tension. This question might seem unwarranted, as how can there be *F*_0_ adjustment when there is no voicing? Continuous adjustment of *F*_0_ regardless of voicing is nonetheless possible if *F*_0_ control and voicing control are relatively independent of each other. The control of fundamental frequency mainly relies on adjusting vocal fold tension by rotating the thyroid cartilage at its joints with the cricoid cartilage (Hollien, 1960), which mainly involves the antagonistic contraction of the CT and the thyroarytenoid (TA) muscles, supplemented with the adjustment of laryngeal height and subglottal pressure by the contraction of the thyrohyoid, sternohyoid, and omohyoid muscles (Atkinson, 1978). Voicing control, on the other hand, is done by abduction and adduction of the vocal folds, which mainly involves the lateral cricoarytenoid (LCA) and the interarytenoid muscles (Farley, 1996; Zemlin, 1968). The relative independence of *F*_0_ and voicing control makes it possible to adjust the tension of the vocal folds even when they are not vibrating.

A further issue is how exactly *F*_0_ contours should be aligned relative to the syllable. It has been shown that the *F*_0_ contour of a syllable in English is a movement toward an underlying pitch target associated with lexical stress as well as other concurrent functions (Fry, 1958; Liu *et al.*, 2013; Xu and Xu, 2005). It is further shown that such target approximation movement is synchronized with the syllable in English (Prom-on *et al.*, 2009; Xu and Prom-on, 2014; Xu and Xu, 2005), just like in Mandarin (Xu, 1998, 1999), i.e., starting from the syllable onset and ending by syllable offset (Xu and Wang, 2001; Xu, 2020).

**FIG. 3.**
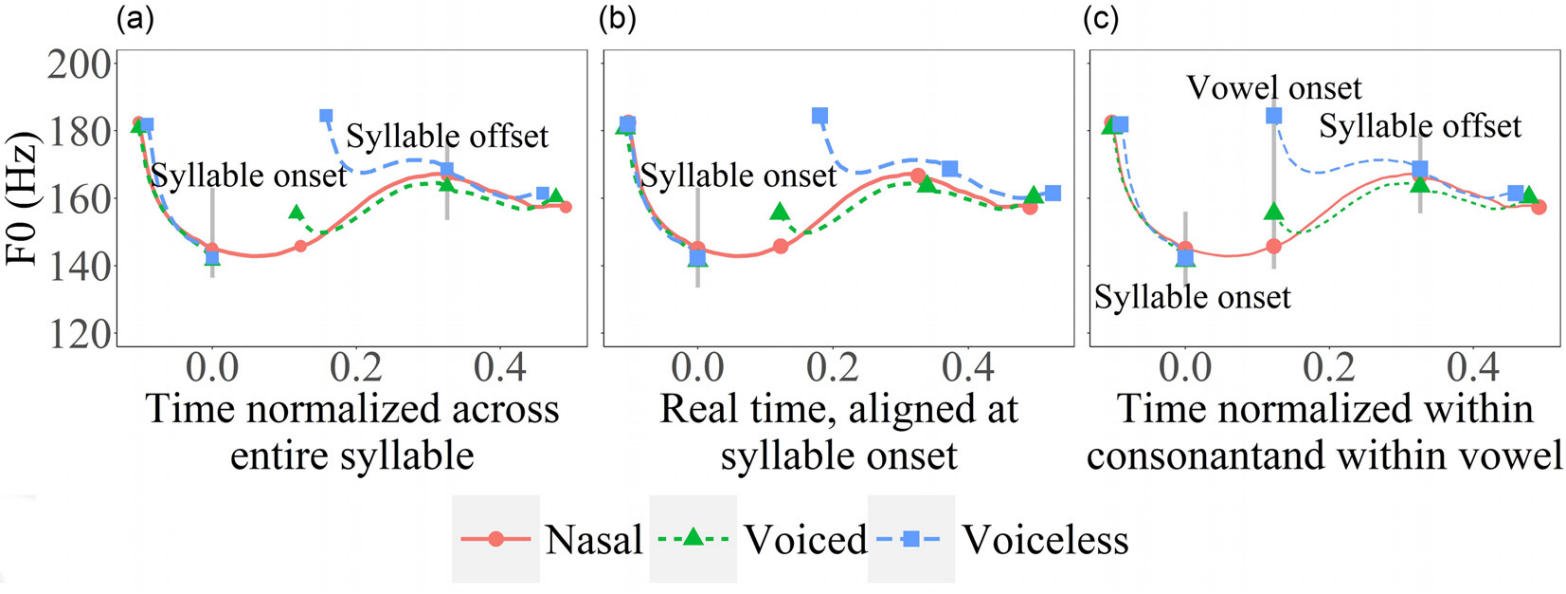
(Color online) Schematic illustrations of different procedures of measuring vertical *F*_0_ perturbation. The curves represent *F*_0_ contours in syllables that start with a nasal consonant (solid), a voiced consonant (dotted), or a voiceless consonant (dashed). In (a), time is normalized across the syllable; in (b) time is actual time, aligned at the syllable onset; and in (c), time is normalized across the consonant closure and the vowel, respectively.

Assuming that the target approaching *F*_0_ movement is indeed synchronized with the syllable in English, the full effect of voice break would be most clearly seen by using sonorant consonants like nasals as the reference, as they allow *F*_0_ to be fully continuous with little vertical perturbation (Xu, 1999; Xu and Xu, 2005). Figure 3 is an illustration based on data from the present study. Here, the solid curve represents the *F*_0_ contour of a syllable with a nasal onset, and the dotted and dashed curves represent those in syllables with voiced and voiceless initial stops, respectively. All the contours are aligned by the onset of the consonant closure on the left and by the offset of the vowel on the right. The time in between is normalized across all the contours. As can be seen, *F*_0_ in both stops starts much later than in the nasal, but they also differ from each other in timing, because voiceless stops have longer voice onset time (VOT) than voiced consonants. What is important is that the estimated vertical perturbation would be different if the alignment of *F*_0_ contours is changed. If the onset of the non-sonorant consonant contours is shifted leftward, the magnitude of the estimated perturbation would increase. Furthermore, if the onset of voiceless consonants is shifted leftward to align with the voiced consonants, the difference between them in perturbation would also increase. Therefore, how *F*_0_ onsets are aligned to each other is a potential confound in the assessment of vertical perturbation.

In previous studies (Chen, 2011; Chen *et al.*, 2017; Lea, 1973; Hombert, 1978; Jun, 1996; Ohde, 1984), including also those that have used nasal consonants as reference (Hanson, 2009; Kirby and Ladd, 2016; Kirby *et al.*, 2020), *F*_0_ contours have always been aligned at the onset of the vowel when estimating *F*_0_ perturbation, as in Fig. 3(c). They differ only in terms of whether there are additional alignment points and whether time-normalization is applied. Some studies applied fixed time windows for the *F*_0_ contours under comparison: 80 ms in Chen (2011), 100 ms in Jun (1996), and 150 ms in Hanson (2009). Instead of fixed time windows, Kirby and Ladd (2016) and Kirby *et al.* (2020) aligned the *F*_0_ contours at vowel onset and offset, and then applied time-normalization across the vowel. The same method was also used by Gao and Arai (2019). By aligning *F*_0_ contours at vowel onset, however, the potential effects of voice break on the assessment of vertical perturbation cannot be seen. Part of the goal of the present study is therefore to find this missing information by considering alternative alignments such as those shown in Figs. 3(a) and 3(b).

A further methodological issue is the quality of *F*_0_ trajectory extraction. The finding of two different kinds of *F*_0_ perturbation in the present study may help to explain the low consensus on the rise-fall dichotomy between voiced and voiceless stops in previous studies. Those that do not catch the initial jumps (House and Fairbanks 1953; Lehiste and Peterson, 1961; Lea, 1973; Hombert *et al.*, 1979; Hanson, 2009) tend to report a simple voicing contrast with *F*_0_ following voiceless stops being higher than the voiced stops. When the initial jumps are preserved, the *F*_0_ falling after both types of consonants is observed (Ohde, 1984; Silverman, 1984; Hanson, 2009^3^). In our statistical comparison of the initial jump of voiced and voiceless stops, the conventional way of *F*_0_ processing that removes the abrupt *F*_0_ shift with trimming and smoothing led to a statistically significant voicing contrast. However, when the initial jump was preserved, the *F*_0_ following voiced and voiceless obstruent consonants was statistically indistinguishable.

### C. The present study

The present study is designed to answer the four critical questions raised in Sec. I by assessing the size and manner of vertical perturbation based on direct comparisons of syllable-wise *F*_0_ contours both before and after the consonant closure. The new approach takes a more careful consideration of alignment and time normalization than has been done before, based on a number of assumptions. First, as discussed in the above section, the adjustment of vocal fold tension should be continuous (rather than in a temporary halt) during the consonant closure. Second, each syllable should have a targeted pitch pattern or pitch target in English as one of its articulatory goals, and this pitch target is associated with word stress as well as other concurrent functions (Fry, 1958; Liu *et al.*, 2013; Xu and Xu, 2005). Second, the *F*_0_ movement toward the pitch targets is fully synchronized with the syllable in English (Prom-on, Xu and Thipakorn, 2009; Xu and Prom-on, 2014; Xu and Xu, 2005) as is in Mandarin (Xu, 1998, 1999).

Another major source of discrepancy in previous reports of perturbation is the technical precision in *F*_0_ extraction. Earlier studies compared *F*_0_ values at a few acoustic landmarks or averaged across a long interval (House and Fairbanks, 1953; Lehiste and Peterson 1961). Later experiments have often used autocorrelation with large smoothing windows to extract *F*_0_ contours (Kingston, 2007; Kirby and Ladd, 2016). These methods are not highly sensitive to brief changes in fundamental frequency. As shown by Ohde (1984), brief pitch spikes can often be found at consonant offsets when *F*_0_ is computed directly from vocal cycles. Those spikes are consistent with the *F*_0_ falls at the voice onset reported by Silverman (1984). When using *F*_0_ extraction algorithms with sizable smoothing windows, the spikes might be missed entirely, or smoothed into the following contour, creating the appearance of a long-lasting perturbation (see Fig. 1). In order to catch any consistent but brief perturbations, there is a need to extract F_0_ directly from vocal cycles, as will be described in Sec. II D.

## II. METHOD

### A. Stimuli

The stimuli (Table I) were chosen to allow variation of a target consonant within a varying linguistic context. Target consonants were nasals, voiced and voiceless fricatives, stops and stop-sonorants, and voiceless affricates. These were embedded in CV syllables, CVC syllables with the first consonant as nasals, and CVCV syllables with the first consonant as either nasals or laterals. The target words were embedded in the carrier sentences “I should say W next time.” and “Should I say W next time?” The carries were chosen to prevent the target consonants from being resyllabified with surrounding contexts (Xu, 1998).

**TABLE I.** Words used as stimuli, in different syllable structures and word length.

	CV		CVC		CVCV	
	Voiceless	Voiced	Voiceless	Voiced	Voiceless	Voiced
Nasal		nay		name		Mamie
Fricative	say	they	mace	nave	Laky	lady
Stop	tay	day	make	Meig	Macy	Maisie
Stop sonorant	tray	dray				
Affricate	Che					

### B. Subjects

Subjects were four women and four men, all residents of New Haven, CT, and mostly students at Yale University. Their ages ranged from 20 to 54 years (from 20 to 24, excluding one subject), and all were native speakers of General American English. One subject, who had no difficulty with the task, had received six months of speech therapy as a young child, to treat a minor lisp. Otherwise, no speech or language disorders were reported.

### C. Recording procedure

The recording was done in a soundproof studio at Haskins Laboratories, New Haven, CT. Subjects sat before a computer screen, on which one stimulus sentence appeared at a time. They read each sentence out loud into a head-mounted microphone and were recorded digitally onto the hard drive of an Apple Macintosh computer. Each sentence was presented five times. To elicit a narrow focus on the target word, we presented it in all capital letters and instructed subjects to emphasize it. Other intonational patterns, noticeable pauses, or voicing anomalies (most commonly creaky voice) rendered some tokens unusable. When this was noticed during the recording, the subject was asked to repeat the sentence. Some problems were not noticed, however, and occasionally both instances of a repeated token turned out to be usable, so the actual number of tokens was in some cases more or less than five.

### D. Pitch extraction and processing

**FIG. 4.**
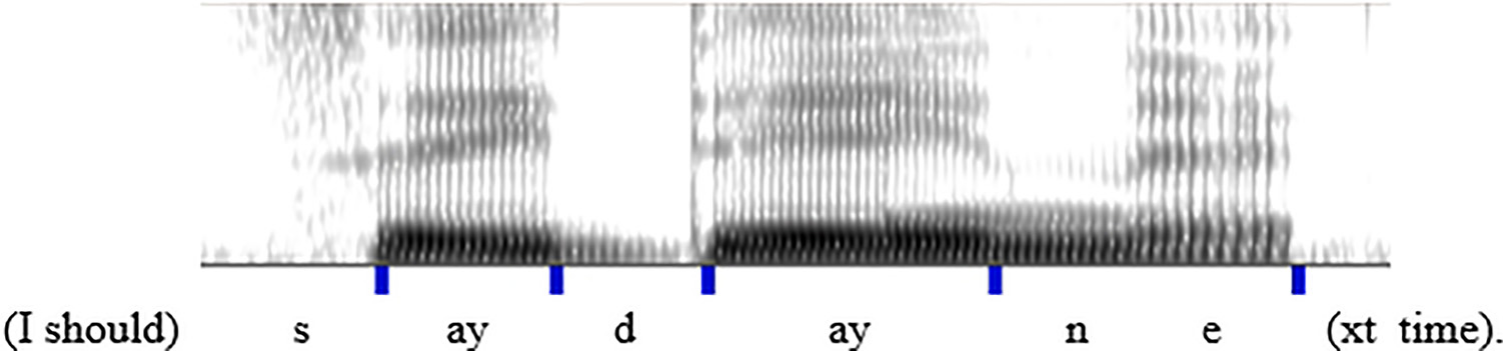
(Color online) An example of segmentation of consonantal and vocalic intervals.

Phonetic data were extracted using a special version of ProsodyPro (Xu, 2013), a Praat (Boersma and Weenink, 2020) script for large-scale analysis of speech prosody. The script first used Praat's To PointProcess function to mark all the vocal cycles. The marked cycles were then manually rectified before being converted to *F*_0_ curves. Segment boundaries were manually labeled at the onset of consonant closure and at the onset of vowel formants in both the target word and part of the carrier (… say __ next…), as illustrated in Fig. 4.

**FIG. 5.**
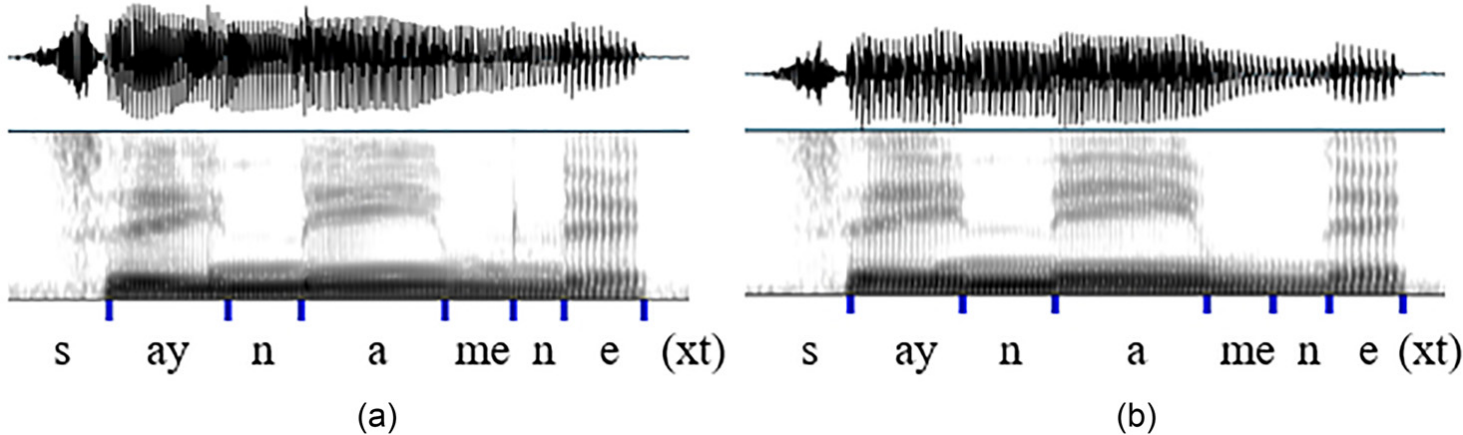
(Color online) (a) An example of a burst at labial release between [m] and [n]. (b) An example of an arbitrary boundary in the middle of a nasal geminate.

In the case of the sentence “I should say name next time,” the boundary between [m] and [n] was not always easy to determine from the waveform or the spectrogram. Sometimes there was a faint burst that accompanied the labial release, and this was marked as the boundary, as shown in Fig. 5(a). Otherwise, the boundary was marked in the center of geminated nasal murmur [Fig. 5(b)].

Further analyses were performed using a custom-written version of ProsodyPro. The *F*_0_ curves were trimmed with an algorithm described in Xu (1999), to remove sharp spikes. The vocal cycle next to a silent interval longer than 33 ms was exempted from this trimming to preserve the sharp spikes that consistently occur at voice onset and offset (based on the assumption that normal *F*_0_ would not go below 30 Hz). The statistical analysis was conducted using linear mixed-effect models by lme4 (Bates *et al.*, 2015) and emmeans (Lenth *et al.*, 2020) for *post hoc* tests in the R (R Core Team, 2020). Random intercepts for SUBJECT and by-SUBJECT random slopes for fixed effects were then incorporated maximally (Barr *et al.*, 2013). Subsequently, potential fixed effects were added. Only fixed effects that were judged to be superior to less specified models tested by likelihood-ratio tests were included in the model.

## III. RESULTS

### A. Graphical comparison of *F*_0_ contours

**FIG. 6.**
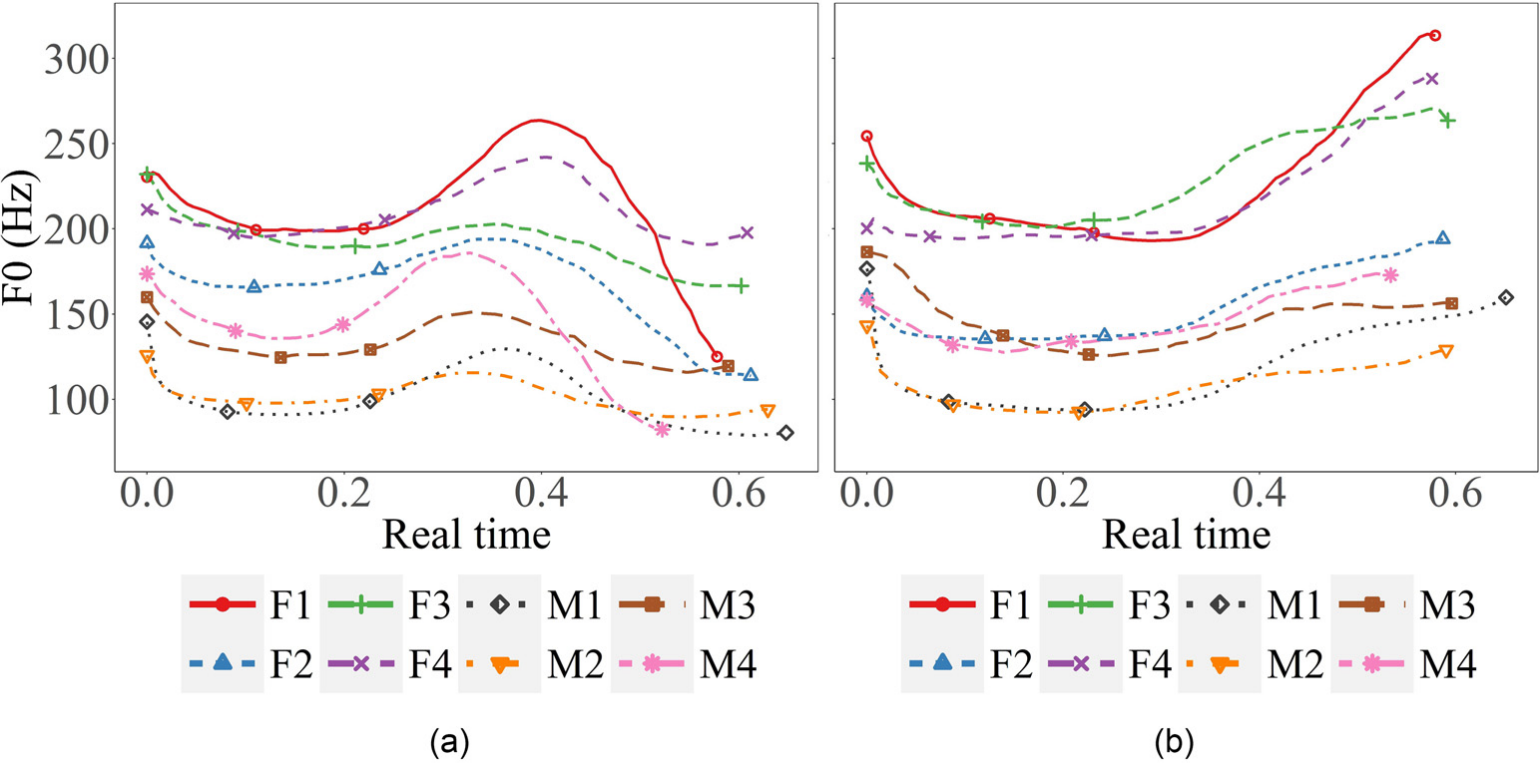
(Color online) (a), (b) Sample mean *F*_0_ contours for the target word “nay” embedded in declarative (left, a) and interrogative (right, b) sentences.

Before deciding what measurements to take for statistical analysis, we first made direct comparisons of the *F*_0_ contours to identify major differences between the conditions. Figure 6 shows examples of mean *F*_0_ contours by individual subjects, with Fig. 6(a) showing those of the target word /nay/ in a statement and Fig. 6(b) in a question. The vertical differences in *F*_0_ are large, with female subjects tending to have higher fundamental frequencies. There are some differences in the location of the *F*_0_ peaks. Regardless of the differences in the vertical level and the peak location, however, all speakers show similar general patterns.

**FIG. 7.**
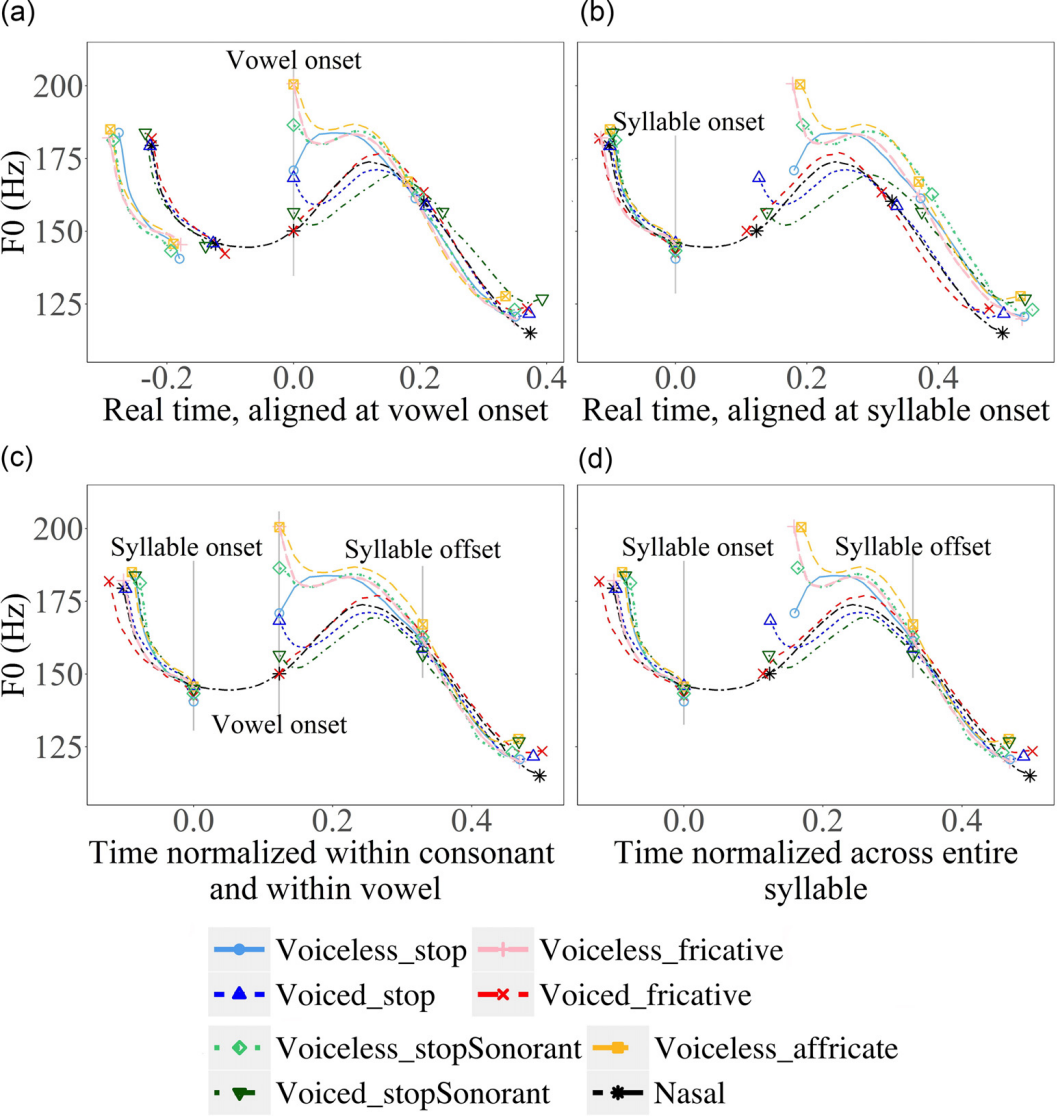
(Color online) (a)–(d). Mean *F*_0_ contours in target CV syllables (also showing parts of the carrier sentence) with different types of consonants in declarative sentences. The methods of alignment and time-normalization are specified below each plot. The vertical lines indicate the alignment points, and the symbolic markers indicate segment boundaries. The consonants having the same manner of articulation are in paired colours with different grayscale values. The voiced consonants are darker than their voiceless counterparts.

**FIG. 8.**
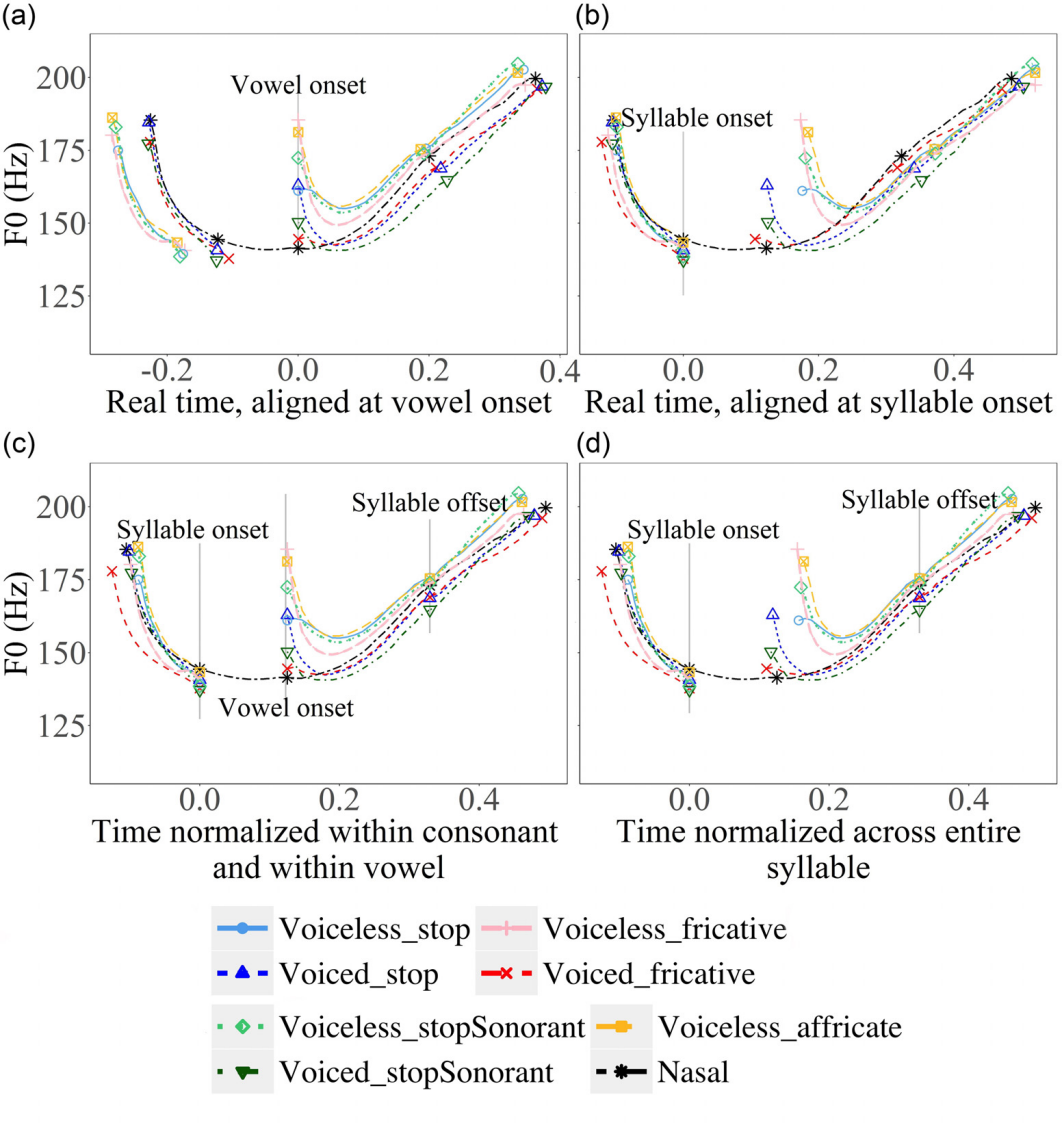
(Color online) (a)–(d) Mean *F*_0_ contours of vowels following target consonants in CV syllables (also showing parts of the carrier sentence) with different types of consonants in interrogative sentences. The methods of alignment and time-normalization are specified below each plot. The vertical lines indicate the alignment points, and the symbolic markers indicate segment boundaries. The consonants having the same manner of articulation are in paired colours with different grayscale values. The voiced consonants are darker than their voiceless counterparts.

**FIG. 9.**
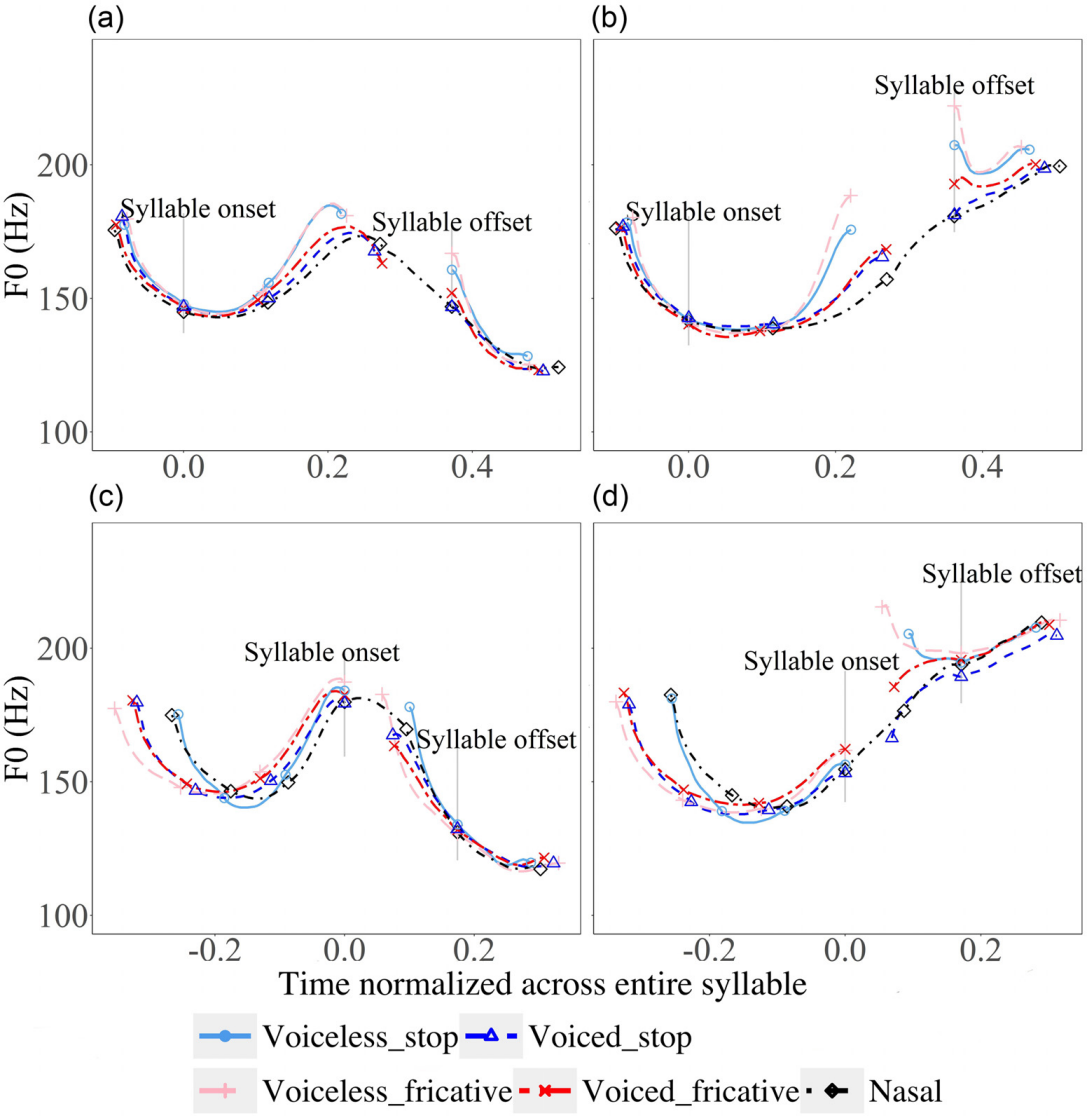
(Color online) Mean *F*_0_ contours of vowels following target consonants in CVC syllables [(a) and (b)] and CVCV [(c) and (d)] and parts of carrier sentences. The time points of consonants are normalized with reference to the mean time points of nasals. Carrier sentence is declarative [left, (a) and (c)] or interrogative [right, (b) and (d)]. The vertical lines indicate the alignment points and the symbolic markers indicate segment boundaries. The consonants having the same manner of articulation are in paired colours with different grayscale values. The voiced consonants are darker than their voiceless counterparts.

Figure 7 shows mean *F*_0_ contours with different ways of alignment and normalization. *F*_0_ of CV syllables and parts of the carrier sentence in statements are aligned at vowel voice onset (a), syllable onset (b), syllable offset (c), and normalized across the entire syllable with alignment at both syllable edges (d). For display purposes only, each contour is an average across all repetitions by all subjects of the given stimulus. When averaging, each segment of each token is sampled at 20 even-spaced points. In the real-time plots, the mean time and *F*_0_ of each of the points were averaged across repetitions and speakers. For the time-normalized plots, the mean time of each type of consonant was recalculated with reference to the mean time of nasals to align these points at both syllable onset and offset. The average plots in Figs. 7–9 reliably represent our data (see the supplementary material^2^ for individual plots for all participants).

In order to establish an appropriate reference level, we plotted *F*_0_ curves using the syllable-wise alignment and conventional alignment methods employed in previous research. As can be seen in Fig. 7, methods of alignment and time-normalization both have clear consequences. When aligned at voice onset [Fig. 7(a)] following previous studies (Lea, 1973; Hombert, 1978; Ohde, 1984; Jun, 1996; Hanson, 2009; Chen, 2011), the *F*_0_ curves of different consonants vary greatly both before and after the consonants. Aligning the *F*_0_ contours at syllable onset [Fig. 7(b)] results in variations at the end of the syllable and the following contexts. When the *F*_0_ contours are aligned at both vowel onset and offset [Fig. 7(c)], as done in Kirby and Ladd (2016), Kirby *et al.* (2020), and Gao and Arai (2019), the amount of cross-consonant *F*_0_ difference is as large as in Fig. 7(a). Time normalizing *F*_0_ curves between the onset and offset of the target syllable [Fig. 7(d)] seems to exhibit the least variable *F*_0_ patterns across consonant types both within the target syllable and in the surrounding carrier sentences. In the following analysis, therefore, we will focus on comparing *F*_0_ contours time-normalized with respect to the syllable.

Looking more closely at Fig. 7(d), we can see that, with the exception of voiced fricative, *F*_0_ is first perturbed upward by non-sonorant consonants relative to the nasal baseline, although there are also apparent differences in voice onset time between various types of consonants. Afterward, for most of the consonant types, *F*_0_ drops sharply toward the nasal baseline and starts to shadow its contour shape for the rest of the syllable. However, for voiceless stops, surprisingly, *F*_0_ first rises rather than falls, and then also starts to shadow the nasal contour. Besides the initial drop or rise, there are also apparent differences between the consonant types in subsequent overall *F*_0_ height, with voiceless consonants generally having higher *F*_0_ than voiced consonants. These height differences, though gradually reducing over time, persist all the way to the end of the vowel.

Figure 8 displays *F*_0_ contours in questions with various alignment and time-normalization schemes. Again, *F*_0_ is perturbed upward after all non-nasal segments, although there is much variation in terms of perturbation size. After this initial jump, like in statements, *F*_0_ quickly drops toward the nasal baseline and starts to shadow its shape for the rest of the syllable duration. Interestingly, voiceless stops again show the smallest perturbation/jump among the voiceless consonants. But unlike in statements, *F*_0_ drops rather than rises after the initial jump. Presumably, the initial jump, though small in size, has raised *F*_0_ much higher than the targeted low F_0_ represented by the nasal contour. Also, like in statements, the overall *F*_0_ height after the initial jump is higher in voiceless consonants than in voice consonants.

Figure 9 shows *F*_0_ contours of CVC [Figs. 9(a) and 9(b)] and CVCV [Figs. 9(c) and 9(d)] syllables with part of the carrier sentences in statements and questions. In both cases, the target consonant is the second consonant in the sequences. These syllables enable the examination of anticipatory effects of obstruent consonants on the preceding *F*_0_ within and across syllable boundaries. For CVC syllables in statements, as can be seen in Figs. 9(a) and 9(b), pre-closure *F*_0_ of non-sonorant consonants inevitably drops sharply after reaching a peak. But before those drops, the overall *F*_0_ height is raised in all cases relative to the nasal baseline. Interestingly, here the consonants seem to be grouped by voicing in statements. Similar overall raising of *F*_0_ height by coda consonants is also seen in questions, except that there are no sharp drops before consonant closure. In contrast, for CVCV syllables, as shown in Figs. 9(c) and 9(d), the *F*_0_ contours of vowels preceding the target consonants do not seem to diverge in both statements and questions. Instead, the lack of the anticipatory effect appears to parallel what we have seen in Figs. 7 and 8 for CV syllables, where the *F*_0_ of vowels in the carrier words converges regardless of the upcoming consonants.

To summarize the graphical comparison, with *F*_0_ contours of nasal consonants as the baseline, a number of initial observations can be made. First, non-sonorant initial consonants seem to exert two kinds of perturbations: (a) an abrupt initial jump in *F*_0_ at voice onset, followed by either a sharp drop or rise (voiceless stop in statement), and (b) a sustained raising (voiceless consonant) or lowering of *F*_0_ height throughout the rest of the syllable. Second, non-sonorant coda consonants also seem to exert two kinds of perturbations: (a) an abrupt drop in *F*_0_ right before voice offset in statements, and (b) a raising of *F*_0_ that extends back toward the midpoint of the vowel. Finally, aspiration, especially in stops, seems to reduce the magnitude of initial jump. This has led to a rise rather than a drop of *F*_0_ immediately after voice onset in a statement. In the next session, we will run statistical tests on the raw data to verify the visual observations.

### B. Statistical analysis

**FIG. 10.**
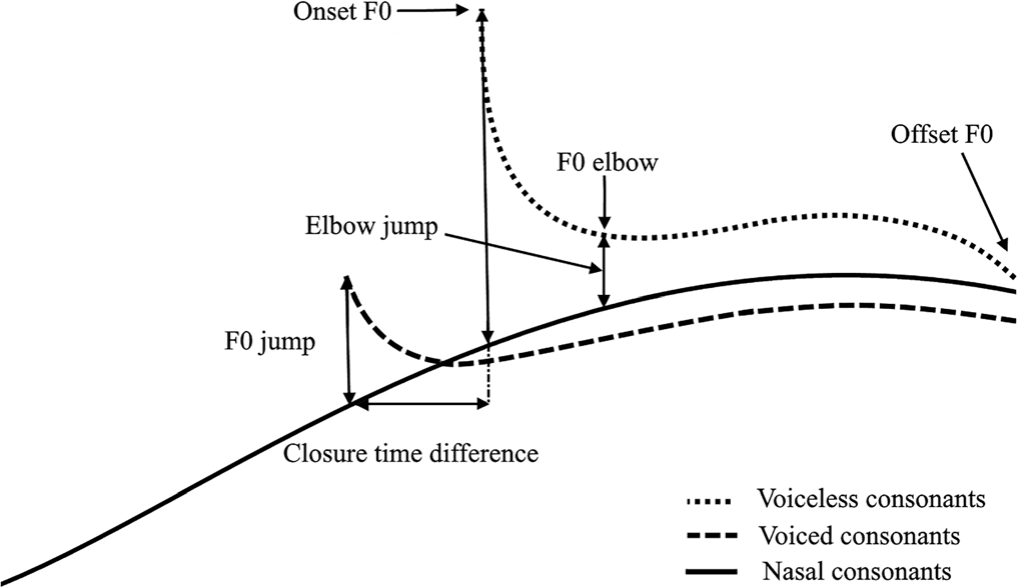
Illustration of onset *F*_0_, *F*_0_ jump, *F*_0_ elbow, elbow jump, and offset *F*_0_.

The graphical comparison of *F*_0_ contours shows initial indication of three different kinds of influences by initial consonants on *F*_0_: (a) a voice break that interrupts continuous *F*_0_, (b) a brief yet sometimes large jump relative to the nasal baseline, and (c) a long lasting raising or lowering effect, also relative to the nasal baseline. To closely examine these influences, closure duration, onset *F*_0_, *F*_0_ jump, *F*_0_ elbow, elbow jump, and offset *F*_0_ of all the repetitions by each speaker were measured and analysed, as illustrated in Fig. 10. For voiceless consonants, the closure duration equals VOT, while for voiced consonants, it is the time elapsed between the oral closure and the onset of the following vowel (thus disregarding any voicing during closure). Onset *F*_0_ is the conventional way of observing initial consonantal perturbation, which is the first *F*_0_ point at the onset of the vowel. *F*_0_ jump is a new measurement not used in previous studies, which indicates the difference between onset *F*_0_ and the *F*_0_ of nasal baseline at the same relative time in normalized time, in the same intonation. Similar to *F*_0_ jump, elbow jump is another new measurement that indicates the difference between *F*_0_ elbow and the *F*_0_ of nasal baseline in the same intonation at the same relative time in normalized time, where *F*_0_ elbow is the *F*_0_ turning point after the initial *F*_0_ jump. Finally, offset *F*_0_ is the *F*_0_ at the end of the vowel preceding a target consonant, which evaluates whether the perturbation effects last until the end of the syllable.

#### 1. Carryover effect

##### a. Consonant closure duration.

As we can see from Figs. 7 and 8, there are noticeable differences in closure time between various classes of consonants, and the shape of *F*_0_ contours at the beginning of the following vowels are influenced by the duration of the closure. The longer the closure, the greater the magnitude of the initial *F*_0_ perturbation, except for voiced stops. Table II lists means and standard deviations of closure duration of consonants in CV syllables separated by consonant types and intonation contexts. For the sake of data balance, statistical analysis was performed only on the stops, fricatives, and stop-sonorants that are minimal pairs. In a set of linear mixed models, CVOICE (voiced, voiceless), CMANNER (stop, fricative and stop-sonorant), INTONATION (statement, question), and their interaction were included as potential fixed effects. CVOICE improves the fit of the model (χ^2^ = 24.077, df = 1, *p <* 0.001): voiceless consonants tend to have longer closures than voiced consonants. CMANNER (χ^2^ = 18.255, df = 2, *p <* 0.001) also significantly predicts closure duration. The *post hoc* comparison showed that stop-sonorants have longer closures than fricatives (*p* < 0.001) and stops (*p* = 0.046). Meanwhile, closure duration of stops is longer than the fricatives (*p =* 0.005). INTONATION (χ^2^ = 2.591, df = 1, *p =* 0.108) does not significantly improve the model. The interaction between CVOICE and CMANNER (χ^2^= 10.861, df = 2, *p =* 0.004) is significant. When the consonant is voiceless, the contrast in closure duration between stops and fricatives is not significant (*p =* 0.895), but the contrast is significant in voiced consonants (*p* = 0.004).

**TABLE II.** Means (standard deviations) of closure duration (ms), onset *F*_0_ (Hz), and *F*_0_ jump (Hz).

Consonant type	Statement			Question		
	Closure duration	Onset F_0_	F_0_ jump	Closure duration	Onset F_0_	F_0_ jump
Nasal	118 (21)	156 (43)	NA	117 (24)	148 (46)	NA
Voiced stop	122 (31)	174 (46)	18 (9)	118 (27)	170 (50)	22 (12)
Voiced fricative	102 (27)	157 (48)	2 (14)	99 (32)	152 (48)	4 (11)
Voiced stop-sonorant	134 (21)	163 (44)	7 (9)	119 (35)	158 (52)	10 (14)
Voiced consonant (excluding nasal)	119 (24)	165 (50)	9 (8)	112 (30)	160 (50)	12 (12)
Voiceless stop	175 (30)	177 (46)	13 (19)	171 (32)	166 (41)	18 (15)
Voiceless fricative	172 (26)	209 (52)	46 (24)	164 (23)	193 (51)	45 (15)
Voiceless stop-sonorant	189 (27)	192 (42)	27 (20)	175 (20)	178 (43)	30 (12)
Voiceless affricate	184 (29)	206 (47)	40 (15)	179 (26)	188 (51)	39 (24)
Voiceless consonant	179 (26)	196 (45)	32 (14)	172 (24)	182 (45)	33 (12)

The realisation of voicing in English consonants is influenced by linguistic contexts such as word position, adjacent consonants, and lexical tones (Davidson, 2016). Table III lists the percentages of phonetically voiced tokens among all phonological voiced consonants. As we can see from the table, there are individual differences in the production of voicing. Voicing is more likely to begin during the constriction for voiced fricatives and voiced stop sonorants compared with voiced stops. Most of the voiced stops are realized as voiceless unaspirated stops (72%), while the percentages of phonetically voiceless fricatives (33%) and stop sonorants (56%) are much lower. In addition, there are individual differences in voicing implementation. One of the speakers (F4) consistently devoiced all the voiced consonants, but the initial perturbation still differs substantially after voiced and voiceless consonants (see supplementary material^2^ for by-speaker plots). For four of the speakers (F2, F3, M3, and M4), *F*_0_ rises after voiceless stops, exhibiting a distinct pattern from other voiceless consonants (see supplementary material^2^ for by-speaker plots).

**TABLE III.** Percentages of phonetically voiced tokens in phonologically voiced stops, fricatives, and stop sonorants.

		F1	F2	F3	F4	M1	M2	M3	M4
Stop	Statement	0	100	0	0	100	0	80	20
	Question	20	60	0	0	60	0	100	20
Fricative	Statement	100	100	100	0	100	100	100	100
	Question	100	100	100	0	100	40	100	100
Stop-sonorant	Statement	20	100	20	0	100	20	100	80
	Question	40	100	20	0	100	20	100	60

##### b. Onset F_0_ and F_0_ jump.

**FIG. 11.**
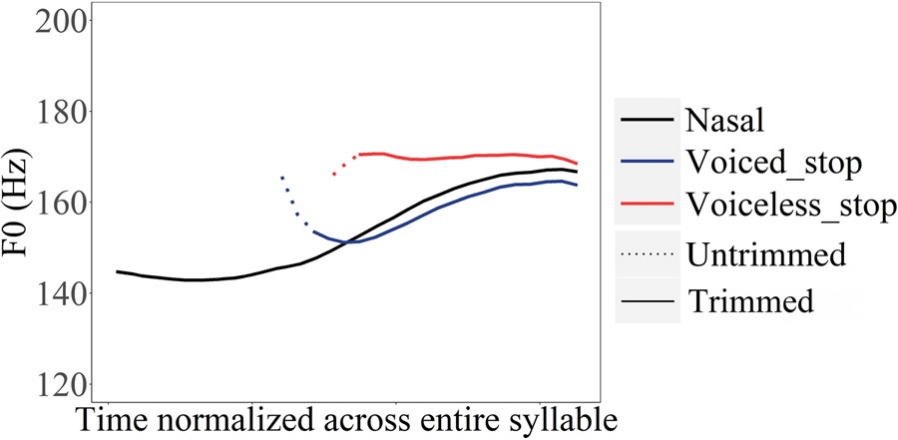
(Color online) Schematic comparisons of *F*_0_ perturbation following voiced and voiceless obstruent consonants when applied with (solid) and without (dotted) trimming and smoothing pitch processing.

As shown in the previous section, closure duration varies with voicing. These variations may affect *F*_0_ at vowel onset, as seen in Figs. 7 and 8. The conventional way of only measuring onset *F*_0_ does not take closure duration into consideration, which may have potentially exaggerated or masked true vertical perturbation. Here, we compare the onset *F*_0_ of stop consonants measured by the conventional pitch-processing method based on autocorrelation with *F*_0_ trimming and smoothing and by our new method (i.e., without trimming and smoothing). As can be seen in Fig. 11, when *F*_0_ trimming and smoothing is applied, the onset *F*_0_ differs by a large amount after voiced stops and voiceless stops. However, when *F*_0_ is obtained without trimming and smoothing, the first few pitch values are very similar regardless of voicing feature.

**FIG. 12.**
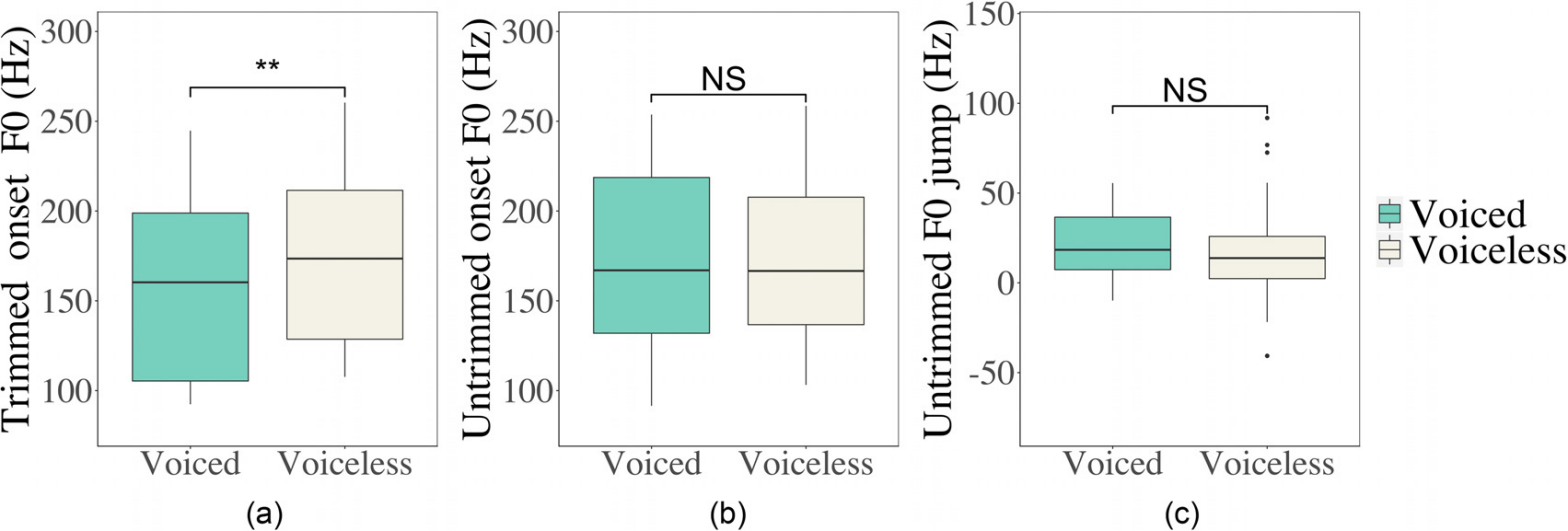
(Color online) Boxplots of trimmed onset *F*_0_ (Hz) (left, a) and untrimmed onset *F*_0_ (Hz) (centre, b) and untrimmed *F*_0_ jump (Hz) (right, c) of vowels following voiced and voiceless stop consonants.

The distributions of the onset *F*_0_ and *F*_0_ jump following voiced and voiceless stops obtained by different pitch processing methods are shown in Fig. 12. A clear distinction of voicing feature can be seen in the trimmed onset *F*_0_, while no such effect is observable in the untrimmed onset *F*_0_ and *F*_0_ jump. We ran statistical tests on the onset *F*_0_ and *F*_0_ jump obtained by the two methods to see whether the pitch extraction and processing method had a significant impact. The main effect of CVOICE is only significant in the model for the trimmed onset *F*_0_ (χ^2^ = 8.386, df = 1, *p =* 0.003) but not for either the untrimmed onset *F*_0_ (χ^2^ = 0.008, df = 1, *p* = 0.930) or the untrimmed *F*_0_ jump (χ^2^ = 0.799, df = 1, *p* = 0.371). The results indicate that the contrast between *F*_0_ following voiced and voiceless is exaggerated when trimming and smoothing are applied.

**FIG. 13.**
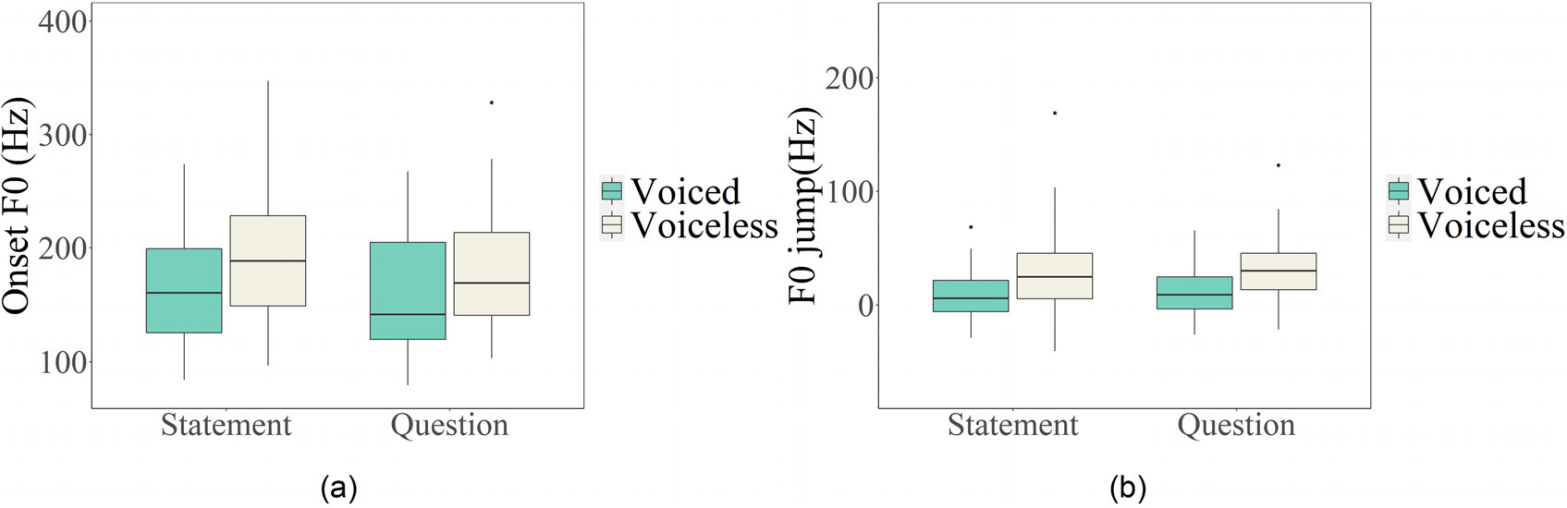
(Color online) Boxplots of onset *F*_0_ (Hz) (left, a) and *F*_0_ jump (Hz) (right, b) of vowels following target consonants across voicing and intonation contexts.

Following the new method, we further evaluated the initial perturbation of other consonant types by measuring both onset *F*_0_ and *F*_0_ jump, as summarized in Table II. As can be seen, the standard derivation (SD) of onset *F*_0_ (SD, 51) is larger than that of *F*_0_ jump (SD, 27) across different conditions. This is further confirmed in Fig. 13, where the boxplots show that *F*_0_ jump is more consistent, i.e., with smaller variance than onset *F*_0_ in both statements and questions, especially for voiceless consonants.

The main effect of CVOICE is significant in the model for onset *F*_0_ (χ^2^ = 10.491, df = 1, *p =* 0.001) and *F*_0_ jump (χ^2^ = 8.398, df = 1, *p =* 0.004). Voiceless consonants show a greater onset *F*_0_ as well as *F*_0_ jump than voiced consonants. In contrast, CMANNER does not seem to have an impact on either onset *F*_0_ (χ^2^ = 4.268, df = 2, *p =* 0.118) or *F*_0_ jump (χ^2^ = 5.016, df = 2, *p* = 0.081). Further, INTONATION is non-significant for either onset *F*_0_ (χ^2^ = 2.664, df = 1, *p =* 0.103) or *F*_0_ jump (χ^2^ = 1.751, df = 1, *p =* 0.186).

**FIG. 14.**
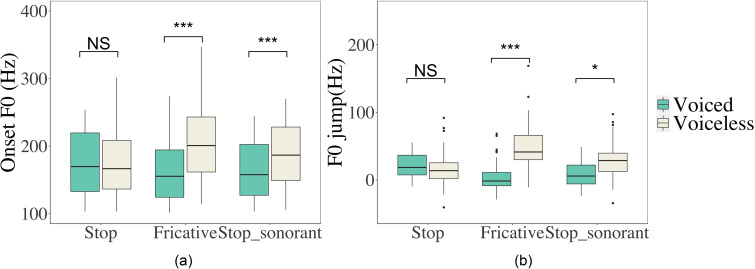
(Color online) Interaction between voicing and manner of articulation in onset *F*_0_ (left, a) and *F*_0_ jump (right, b). Nasals and affricates are excluded.

The interaction between CVOICE and CMANNER is significant for both onset *F*_0_ (χ^2^ = 102.260, df = 4, *p <* 0.001) and *F*_0_ jump (χ^2^ = 104.950, df = 4, *p* < 0.001). As demonstrated in Fig. 14, the voicing contrast is more salient in fricatives (onset *F*_0_: *p <* 0.001; *F*_0_ jump: *p <* 0.001) and stop-sonorants (onset *F*_0_: *p <* 0.001; *F*_0_ jump: *p =* 0.012) than in stops (onset *F*_0_: *p = 1.000*; *F*_0_ jump: *p =* 0.968). It is worth noting that the interaction between CVOICE and INTONATION is significant in the model for onset *F*_0_ (χ^2^ = 8.136, df = 2, *p =* 0.017), whereas *F*_0_ jump is not affected by the interaction (χ^2^ = 1.751 df = 1, *p* = 0.186). As seen in Fig. 13, the onset *F*_0_ of voiceless consonants is marginally higher in statements than questions (*p* = 0.097), but that of voiced stops is similar across intonation (*p =* 0.786). For *F*_0_ jump, which results from subtraction of the nasal baseline from onset *F*_0_, the interference from the interaction between voicing and intonation is eliminated.

**FIG. 15.**
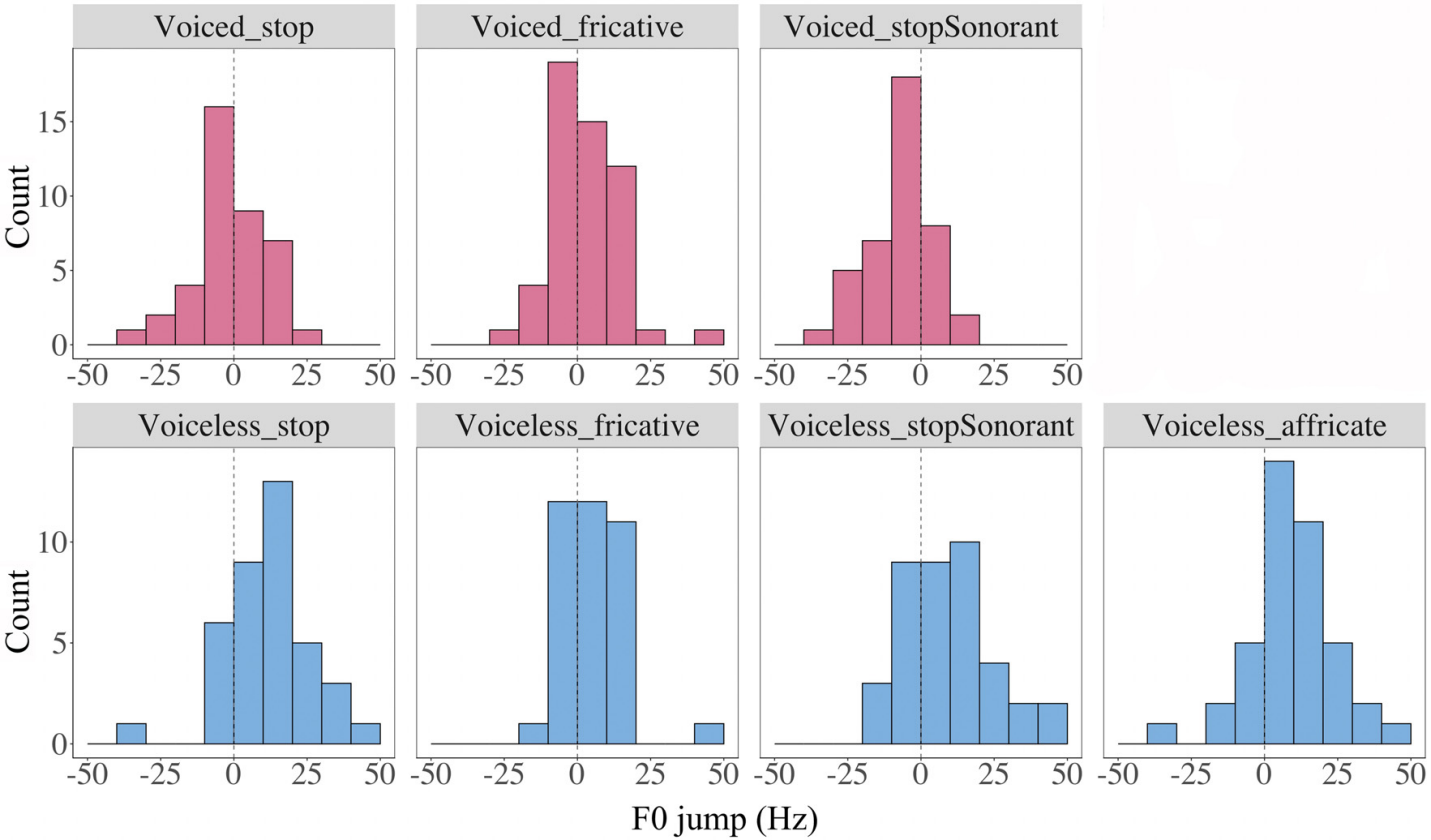
(Color online) Histographic distributions of *F*_0_ jump values by consonant type. The upper panel shows distributions of *F*_0_ jump for voiced consonants and the lower panel for voiceless consonants. In each plot, the dashed vertical line marks the zero point on the *x* axis.

What remains unclear is whether the voicing contrast in the initial perturbation is due to *F*_0_ raising by voiceless consonants or *F*_0_ lowering by voiced consonants. We plotted a histogram of *F*_0_ jump for all consonant types in Fig. 15. As can be seen, except for voiceless stops, nearly all the *F*_0_ jumps of voiceless consonants are above zero, which suggests a significant *F*_0_ raise relative to nasals. And, interestingly, *F*_0_ jumps in voiced stops are also distributed largely above zero. In contrast, voiced fricatives and voiced stop-sonorants contain both negative and positive values. This indicates that voiced stops significantly raise *F*_0_ at vowel onset relative to the nasal baseline, just like voiceless consonants, which is consistent with the findings of Ohde (1984) and Silverman (1984). In other words, instead of *F*_0_ lowering versus *F*_0_ raising, voiced and voiceless stops differ only in the magnitude of *F*_0_ raising as far as *F*_0_ jumps are concerned.

##### c. F_0_ elbow and elbow jump.

**FIG. 16.**
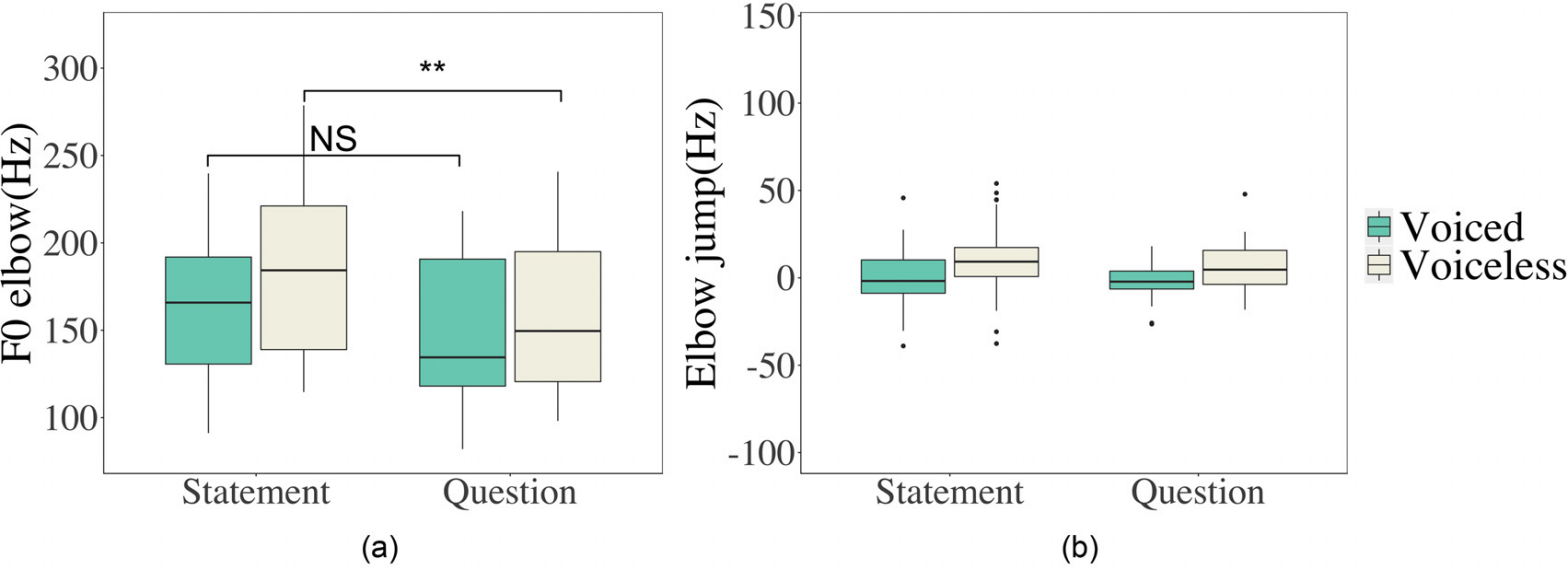
(Color online) Boxplots of *F*_0_ elbow (a) and elbow jump (b) separated by consonant voicing and intonation context. See Fig. 10 for definitions of *F*_0_ elbow and elbow jump.

As can be seen in Figs. 7 and 8, the initial *F*_0_ jump does not last long and the *F*_0_ trajectories of different consonants gradually converge toward the nasal baseline after a sharp turn. The turning point (*F*_0_ elbow) occurs around 41 ms (SD = 22) after vowel onset. However, it is not the case that an *F*_0_ elbow occurs after vowel onset in every utterance. The count and the height of *F*_0_ elbow and elbow jump (the difference between *F*_0_ elbow and the *F*_0_ of nasal baseline in the same intonation at the same relative time point in normalized time, cf. Fig. 10) are summarized in Table IV. Figure 16 shows values of *F*_0_ elbow and elbow jump in different voicing and intonation conditions. Like in the case of onset *F*_0_ and *F*_0_ jump, more variances can be seen in *F*_0_ elbow (SD = 45) than in elbow jump (SD = 15). We fitted separate models for *F*_0_ elbow and elbow jump with CVOICE (voiced, voiceless), CMANNER (stop, fricative, stop-sonorant), INTONATION (statement, question), and their interactions as potential fixed effects. The main effect of CVOICE is significant on *F*_0_ elbow (χ^2^ = 17.339, df = 1, *p <* 0.001) and elbow jump (χ^2^ = 9.270, df = 1, *p* = 0.002): Voiceless consonants have higher *F*_0_ elbow values than voiced consonants. CMANNER does not improve the fit of the model for either *F*_0_ elbow (χ^2^ = 0.442, df = 2, *p =* 0.801) or elbow jump (χ^2^ = 0.348, df = 2, *p =* 0.175). *F*_0_ elbow differs across intonation patterns (χ^2^ = 6.406, df = 1, *p* = 0.011): higher in declarative sentences than in interrogative sentences. In contrast, INTONATION does not significantly predict elbow jump (χ^2^ = 1.074, df = 1, *p* = 0.3). Similar to the results of onset *F*_0_ and jump *F*_0_ presented earlier, the interaction between CVOICE and INTONATION significantly improves the fit of the model for *F*_0_ elbow (χ^2^ = 6.806, df = 1, *p =* 0.009) but not for elbow jump (χ^2^ = 1.271, df = 2, *p =* 0.530). The *F*_0_ elbow of voiceless consonants has higher values in statements than in questions (*p* = 0.002), but not for voiced consonants (*p* = 0.082) (see Fig. 16).

**TABLE IV.** The number of *F*_0_ elbow/total available tokens and means (standard deviations) (in Hz) by intonational patterns and consonant types.

Consonant type	Statement			Question		
	Count	F_0_ elbow	Elbow jump	Count	F_0_ elbow	Elbow jump
Voiced stop	22(40)	161(42)	1(14)	18(39)	139(35)	−4(10)
Voiced fricative	26(40)	161(41)	6(13)	27(40)	144(41)	0(10)
Voiced stop-sonorant	17(38)	167(39)	−13(13)	24(39)	150(45)	−1(6)
Voiced consonants (excluding nasal)	65(118)	163(40)	0(15)	69(118)	145(41)	−1(9)
Voiceless stop	21(40)	188(50)	13(17)	17(37)	157(37)	9(10)
Voiceless fricative	21(39)	160(39)	8(12)	16(40)	144(44)	−1(7)
Voiceless stop-sonorant	25(38)	184(43)	8(16)	14(39)	163(43)	11(16)
Voiceless affricate	29(38)	196(47)	12(18)	13(40)	162(41)	7(13)
Voiceless consonants	96(155)	183(46)	10(16)	60(156)	156(41)	6(13)

**FIG. 17.**
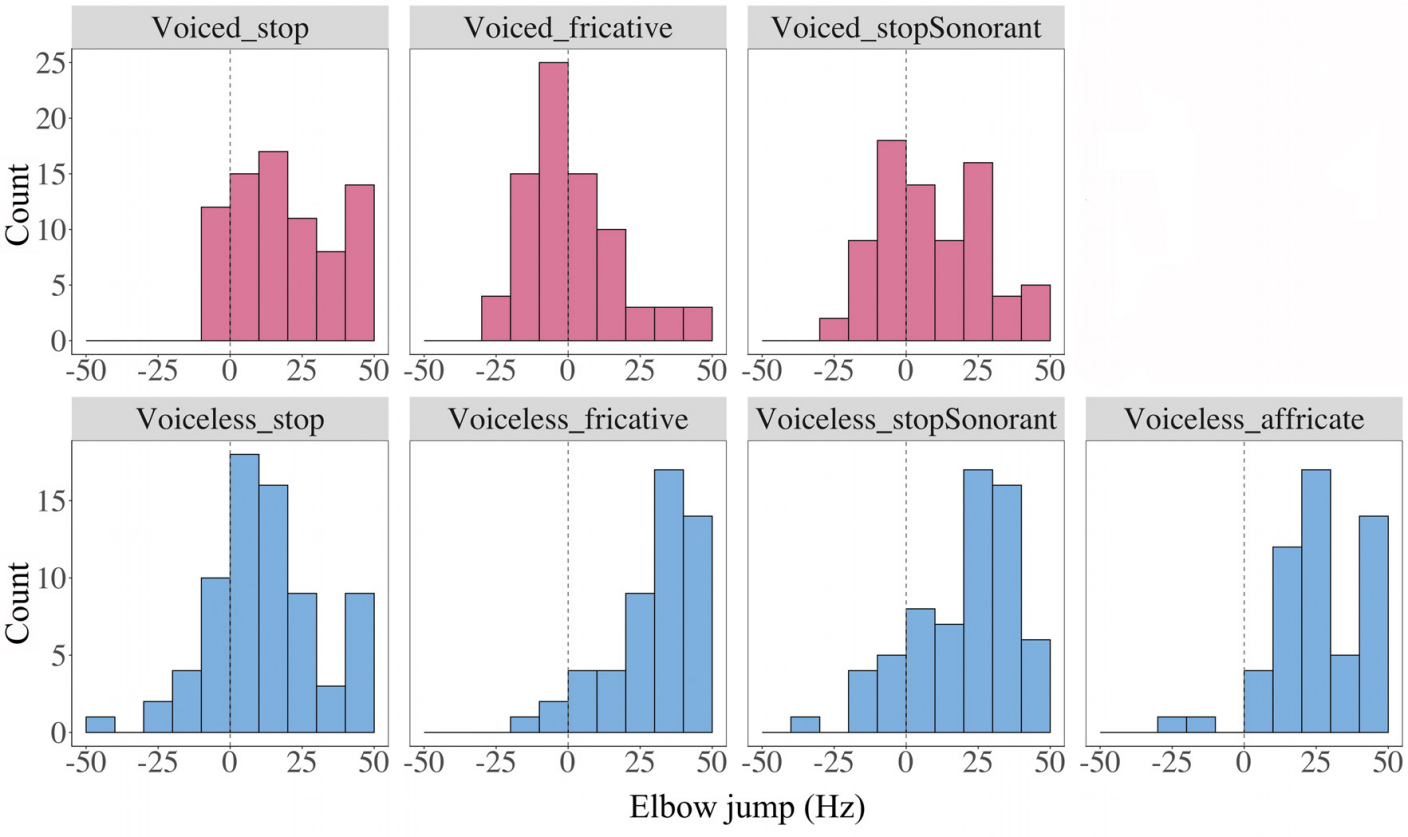
(Color online) Histographic distributions of elbow jump values by consonant type. The upper panel shows distributions of elbow jump for voiced consonants and the lower panel for voiceless consonants. In each plot, the dashed vertical line marks the zero point on the *x* axis.

Figure 17 shows the values of elbow jump for each consonant type. Even after the abrupt initial *F*_0_ jump, there are still clear differences between the *F*_0_ values after voiced and voiceless consonants. Compared with the distribution of *F*_0_ jump (Fig. 15), the raising effects by voiceless consonants have reduced while the lowering effects of voiced consonants have become more evident.

##### d. Offset F_0_.

As seen in Figs. 7 and 8, the differences in *F*_0_ across consonant types do not end by the *F*_0_ elbows but are sustained through the rest of the syllable. Remarkably, what can also be noticed is that the divergence in offset *F*_0_ between voiced and voiceless consonants is not only due to the upward *F*_0_ shifts following voiceless consonants but also due to the downward *F*_0_ shifts following voiced consonants. Means and standard deviations of offset *F*_0_ under different conditions are provided in Table V. Offset *F*_0_ following voiced consonants is considerably lower than the nasal baseline, whereas it is close to the nasal baseline following voiceless consonants. We ran a series of linear mixed models to test whether the voicing contract remains statistically significant by the end of the syllable. CVOICE (voiced, voiceless) improves the fit of the model (χ^2^ = 6.654, df = 1, *p* = 0.010): The offset *F*_0_ of vowels following voiceless consonants is higher than the ones following voiced consonants. However, neither CMANNER (stop, fricative, stop-sonorant: χ^2^ = 3.365, df = 2, *p =* 0.186) nor INTONATION (statement, question: χ^2^ = 1.367, df = 1, *p* = 0.242) shows significant effects on the offset *F*_0_. The results, therefore, indicate that the *F*_0_ height difference due to voicing lasts until the end of the syllable.

**TABLE V.** Means (standard deviations) of offset *F*_0_ (Hz) following different types of consonants in declarative and interrogative carrier sentences.

Consonant type	Statement	Question
Nasal	168(61)	181(51)
Voiced stop	164(55)	176(48)
Voiced fricative	169(59)	178(52)
Voiced stop-sonorant	161(56)	172(46)
Voiced consonants (excluding nasals)	164(56)	176(47)
Voiceless stop	168(60)	183(49)
Voiceless fricative	168(60)	182(52)
Voiceless stop-sonorant	168(59)	183(53)
Voiceless affricate	173(62)	184(53)
Voiceless consonants	169(60)	183(52)

#### 2. Anticipatory effect

##### a. Effect of syllable boundary.

The consonantal perturbation may impact not only the *F*_0_ of the following vowel but also the preceding vowel. As shown in Figs. 9(a) and 9(b), *F*_0_ contours of vowels preceding the coda consonants in CVC syllables do not converge. In contrast, vowels before the target consonants in CV syllables have very close *F*_0_ values (Figs. 7 and 8), which is similar to the first vowels in CVCV syllables where the second consonant is an obstruent, as shown in Figs. 9(c) and 9(d). The means and standard deviations of *F*_0_ offset for vowels in CVC syllables, the first vowels in CV and CVCV syllables are listed in Table VI. We performed statistical analysis on the vowel offset *F*_0_ with CVOICE (voiced, voiceless), CMANNER (stop, fricative), INTONATION (statement, question), and their interaction as potential fixed effects. In CVC syllables, the main effect of CVOICE (χ^2^ = 10.018, df = 1, *p =* 0.002) is significant. The *F*_0_ at the vowel offset is higher when preceded by voiceless consonants than by voiced consonants. Neither CMANNER (χ2 = 1.172, df = 1, *p* = 0.279) nor INTONATION (χ2 = 1.061, df = 1, *p* = 0.303) significantly predicts the offset *F*_0_. The interaction CMANNER and INTONATION (χ^2^ = 21.760, df = 2, *p <* 0.001) is significant: the contrast between stops and fricatives is more pronounced in questions (*p <* 0.001) than in statements (*p =* 0.095). In short, voicing and manner of articulation of coda consonants influence the *F*_0_ of vowels right before the closure and the effect interacts with sentence intonation.

**TABLE VI.** Means (standard deviations) of offset *F*_0_ (Hz) of vowels in CVC syllables, first vowels in CVCV syllables before syllable boundaries and first vowels in CV syllables before word boundaries in declarative and interrogative sentences.

Consonant type	Statement			Question		
	CV	CVC	CVCV	CV	CVC	CVCV
Nasal	152(45)	175(53)	190(52)	150(45)	171(52)	166(51)
Voiced stop	152(42)	167(52)	191(50)	147(46)	176(50)	165(47)
Voiced fricative	148(43)	162(58)	191(53)	145(47)	180(52)	174(50)
Voiced stop-sonorant	151(45)	NA	NA	142(40)	NA	NA
Voiced consonants (excluding nasal)	150(43)	164(55)	191(51)	145(44)	178(51)	169(49)
Voiceless stop	147(44)	190(59)	188(51)	146(45)	180(54)	164(47)
Voiceless fricative	152(46)	182(52)	194(52)	150(49)	199(56)	169(49)
Voiceless stop-sonorant	149(42)	NA	NA	144(41)	NA	NA
Voiceless affricate	152(47)	NA	NA	150(47)	NA	NA
Voiceless consonants	150(44)	186(55)	191(51)	148(45)	190(55)	167(48)

When the syllable boundary is not a word boundary, as in the case of offset *F*_0_ in the first vowel of the CVCV syllable, the main effects of CMANNER (χ^2^ = 5.507, df = 1, *p =* 0.019) and INTONATION (χ^2^ = 5.905, df = 1, *p =* 0.015) are significant, while the main effect of CVOICE (χ^2^ = 0.227, df = 1, *p* = 0.634) is not. No trace of *F*_0_ differences at vowel offset before voiceless and voiced consonants was observed before syllable boundaries.

For vowel *F*_0_ offset preceding CV syllables, when the syllable boundary between the target consonant and the preceding vowel is also a word boundary, the main effect of CVOICE (χ^2^ = 0.056, df = 1, *p =* 0.814), CMANNER (χ^2^ = 0.728, df = 2, *p =* 0.695) and INTONATION (χ^2^ = 0.779, df = 1, *p =* 0.378) are not significant, and neither are the two-way interactions and three-way interactions. The anticipatory *F*_0_ perturbation is also missing here, just like in CVCV syllables. If we combine the findings of offset *F*_0_ in vowels before obstruent consonants in the CV, CVC, and CVCV syllables, it seems clear that anticipatory *F*_0_ modulation at vowel offset is only present within a syllable.

##### b. Time course of anticipatory F_0_ perturbation in CVC syllables.

**FIG. 18.**
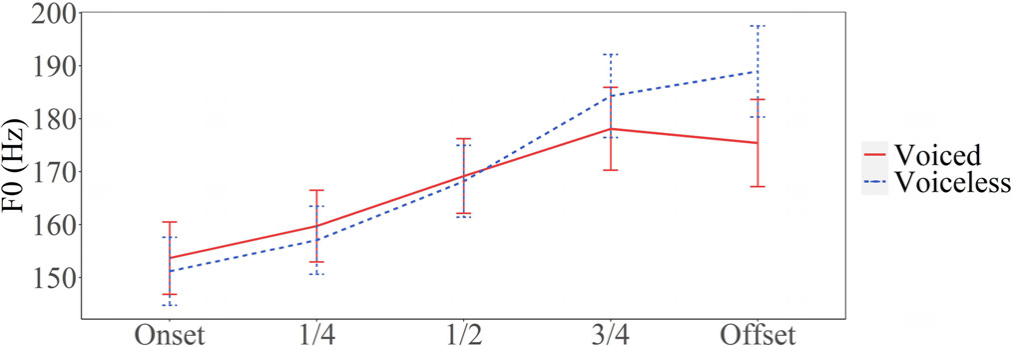
(Color online) *F*_0_ at five relative locations in the vowels preceding voiced consonants (nasals excluded) and voiceless consonants. Error bars show the standard errors.

As seen in Figs. 9(a) and 9(b), in CVC syllables, *F*_0_ contours vary visibly with different types of coda consonants. The differences are the greatest right before the consonant closure, which then gradually reduce leftward and eventually converge to the nasal baseline. Figure 18 plots the time course of the anticipatory *F*_0_ perturbation effect in vowels preceding voiced and voiceless consonants in five in-syllable positions. We can see that *F*_0_ is higher preceding voiceless consonants than preceding voiced consonants. The closer to the target consonant, the more prominent the contrast is. To examine the time course of the anticipatory effect, we fitted linear mixed models with TIME (five levels: onset, 1/4, 1/2, 3/4 of the vowel duration, and offset) being incorporated as a potential categorical fixed effect. In addition, CVOICE (voiced, voiceless), CMANNER (stop, fricative, stop-sonorant), INTONATION (statement, question), and their interactions are included as potential fixed effects. Detailed results of the linear mixed models can be found in Appendix A. The interaction between CVOICE and TIME is significant (χ^2^ = 72.277, df = 4, *p* < 0.001). *Post hoc* comparisons show that the difference in the *F*_0_ of vowels before voiced and voiceless consonants is significant only at the very end of the syllable (*p* < 0.001), but not at the beginning (*p* = 0.995), 1/4 (*p* = 0.990), 1/2 (*p =* 1.000), or 3/4 (*p =* 0.181) of the vowel duration. Overall, the results indicate that there is an anticipatory *F*_0_ perturbation effect that emerges from the very end of the vowel.

## IV. DISCUSSION

The present study aims at achieving an accurate assessment of the nature and scope of the consonantal perturbation of *F*_0_ by testing a number of methodological measures: (1) applying a nasal baseline as the reference; (2) using syllable-wise time-normalization to align *F*_0_ contours in different syllable structures; (3) calculating *F*_0_ cycle-by-cycle without smoothing with a large window; and (4) controlling underlying intonation in carriers spoken as either statements or questions. With these methods, we have found evidence that there are two rather different types of perturbations. One is a brief, yet sometimes large, *F*_0_ jump at the vowel onset relative to the nasal baseline, and the other is a long-lasting raising or lowering of *F*_0_ that persists all the way to the end of the syllable. In addition, we have also observed a brief anticipatory perturbation of F_0_ before a coda consonant.

### A. Large brief perturbations

From Figs. 7(d) to Fig. 8(d), we can see that the initial *F*_0_ at vowel onset is in most cases well off the nasal baseline. We measured this initial deviation of *F*_0_ in two different ways: onset *F*_0_ (absolute *F*_0_) and *F*_0_ jump (relative to nasal baseline). Statistical results show a significant effect of consonant voicing on both onset *F*_0_ and *F*_0_ jump, but no effect of manner of consonant articulation. Onset *F*_0_ is more variable than *F*_0_ jump as a consequence of the impact of the interaction between consonant voicing and sentence intonation (see Fig. 13). The onset *F*_0_ values of voiceless consonants are higher in statements than in questions. After this jump, in each case, *F*_0_ quickly turns toward a trajectory that shadows the nasal baseline for the rest of the syllable. Despite the shadowing, in most cases, the long-term trajectories stay away from the nasal baseline, with the general tendency of higher *F*_0_ after voiceless consonants and lower *F*_0_ after voiced consonants. Thus, the initial jumps seem to be rather different from the longer-lasting effects. Figures 7(d) and 8(d) further show that, surprisingly, *F*_0_ jump is much smaller after voiceless stops than after other voiceless consonants. In Fig. 7(d), after the release of a voiceless stop, *F*_0_ even rises up to join the cluster of voiceless trajectories that are elevated well above the nasal baseline (which, as mentioned in Sec. III B 1 a, occurred in four of the eight speakers). This further implies that the initial jump is likely due to a different mechanism from the longer-term effects.

The first possibility is that the initial *F*_0_ jump is due to an aerodynamic effect (Ladefoged, 1967). In that hypothesis, the buildup of oral pressure during a voiced stop reduces the pressure drop across the vocal cords, thus decreasing *F*_0_ in the following vowel. In a voiceless stop, especially if it is aspirated, the high transglottal airflow at the release creates a boosted Bernoulli force, leading to increased *F*_0_ in the following vowel (Hombert *et al.*, 1979). However, the present data show that large *F*_0_ jumps occur after the release of both voiced and voiceless obstruents. Moreover, at even greater odds with the aerodynamic hypothesis, voiceless stops show much smaller *F*_0_ jumps than the other voiceless obstruents (Table II). This goes against the finding of Löfqvist *et al.* (1995) that the level of airflow is greater after a voiceless stop than after a voiced stop.

Another possibility is that much of the *F*_0_ jump could be due to a brief falsetto vibration (Xu, 2019). That is, the initial vibration at voice onset after an obstruent may involve only the outer (mucosal) layer of the vocal folds (Titze, 1994), which has a higher natural frequency than the main body of the vocal folds, due to its smaller mass (Miller *et al.*, 2002). At the moment of voice onset, transglottal airflow is going through a sharp drop as the vocal folds are quickly being adducted for voicing. The adduction process has to first involve the outer layers of the folds before engaging the main body, and a vibration involving only the outer layer would generate *F*_0_ at the falsetto register rather than the chest register (Titze, 1994). Falsetto vibration has been suggested to happen at the end of utterance offsets, where *F*_0_ is often observed to jump up abruptly in breach of the on-going downward intonation contour (Xu, 2019). This brief falsetto vibration hypothesis would predict that the level of *F*_0_ jump is related to the speed of vocal fold adduction at voice onset, as falsetto vibration is more likely to happen when the adduction speed is relatively slow. This would be the case in voiceless fricatives which likely requires precise control of transglottal airflow. As shown in Table II, voiceless fricatives indeed have the largest *F*_0_ jumps in both statements and questions. The brief falsetto vibration hypothesis would also predict that the magnitude of *F*_0_ jump can vary positively with boundary strength. We analyzed the *F*_0_ following the medial consonant in CVCV syllables (see Appendix B for the descriptive statistics and Appendix C for the results of the linear mixed models). Compared with the initial consonant at the word boundary in CV syllables, the closure duration of the medial consonant is much shorter and the magnitude of *F*_0_ jump is also smaller in CVCV syllables.

**FIG. 19.**
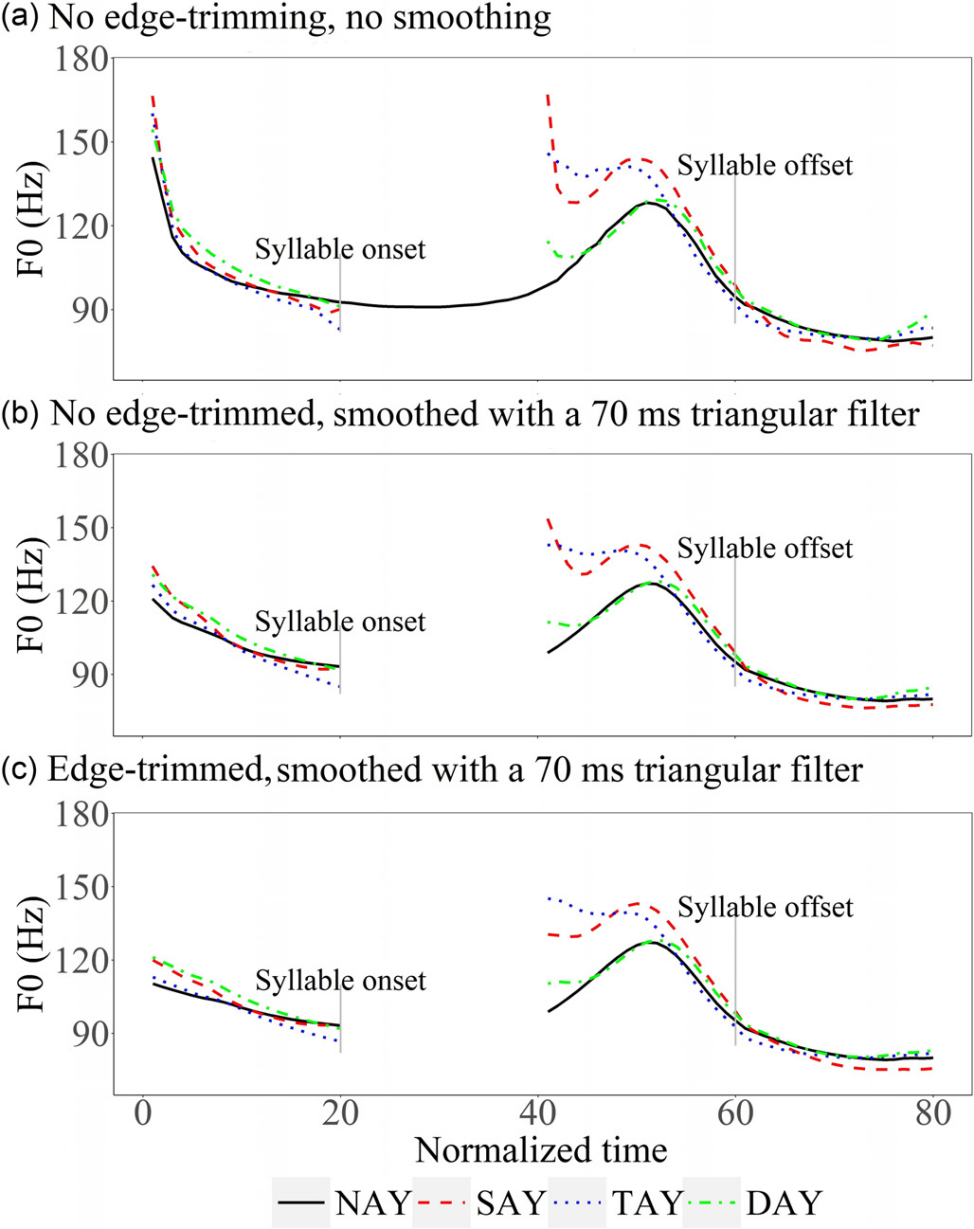
(Color online) Illustration of *F*_0_ curves obtained by various trimming methods.

The brevity of the initial *F*_0_ jump makes it tricky to capture in *F*_0_ analysis, however, as illustrated in Fig. 19. All the *F*_0_ contours in the figure were generated by taking the inverse of every vocal period to obtain the raw *F*_0_, and then applying a trimming algorithm (Xu, 1999) to prune very local spikes. They differ only in (a) whether the trimming is applied across silent intervals (edge-trimmed), and (b) whether a smoothing filter is applied after trimming. In Fig. 19(a), trimming was not applied across silent intervals longer than 33 ms (i.e., when *F*_0_ would go below 30 Hz). With this method (which was used in the present study), the large F_0_ jumps (relative to the nasals) as well as the sharp drops are clearly visible. In Fig. 19(b), trimming was again not applied across silent intervals, but a 70-ms triangular filter was applied to smooth the raw *F*_0_. As a result, the initial jumps and the following drops are now much smaller. In Fig. 19(c), trimming was applied across silent intervals before smoothing. As can be seen, the large *F*_0_ drops have now mostly disappeared, although the *F*_0_ jumps are still clearly visible. With the new method, the large initial *F*_0_ jumps can be found for all the speakers, despite some differences in magnitude (see supplementary material^2^ for by-speaker plots).

The finding of two different kinds of *F*_0_ perturbation in the present study may help to explain the low consensus on the rise-fall dichotomy between voiced and voiceless stops in previous studies. Those that do not catch the initial jumps (House and Fairbanks, 1953; Lehiste and Peterson, 1961; Lea, 1973; Hombert *et al.*, 1979) tend to report a simple voicing contrast with *F*_0_ following voiceless stops being higher than the voiced stops. When the initial jumps are preserved, the *F*_0_ fall after both types of consonants is observed (Ohde, 1984; Silverman, 1984; Hanson, 2009). In our statistical comparison of the initial jump of voiced and voiceless stops, the removal of the abrupt *F*_0_ shift with trimming and smoothing led to a statistically significant voicing contrast. When the initial jump was preserved, however, the *F*_0_ following voiced and voiceless obstruent consonants was statistically indistinguishable.

The present data also show that the brief perturbation lasts only around 41 ms (SD = 22), after which there is frequently a turning point where the initial perturbation fades away and the *F*_0_ of all consonants starts to shadow the nasal baselines. At the *F*_0_ turning point (*F*_0_ elbow and elbow jump), voiceless consonants show higher absolute *F*_0_ than voiced consonants, and the difference is more prominent in statements than in questions [Fig. 16(a)]. When measured in terms of elbow jump, which is relative to the nasal baseline, *F*_0_ shows less variance and is not influenced by the sentence intonation [Fig. 16(b)]. Again, similar to the case of onset *F*_0_ versus *F*_0_ jump, voicing contrast at the *F*_0_ turning point, though large in magnitude, is masked by sentence intonation due to greater variability than elbow jump. The syllable-wise alignment with the nasals eliminates the interference of intonation, which leads to higher consistency in F_0_ jump and elbow jump.

### B. Sustained carryover perturbation

After the *F*_0_ turning point, a smaller upward perturbation is still evident when comparing voiceless consonants with voiced consonants. This effect has a magnitude of around 8 Hz, and it progressively diminishes till the end of the syllable. Furthermore, the distribution of this effect is different from that of the larger initial effect. While the former shows varying magnitudes after different obstruent consonants, the latter shows little differences in magnitude between consonants. This latter effect is consistent with the vocal fold tension mechanism proposed by Halle and Stevens (1971). That is, in a voiceless obstruent the vocal folds are stiffened to impede glottal vibration during the consonant closure, while in a voiced obstruent the vocal folds are slackened to facilitate glottal vibration. Previous studies, however, have not been able to find clear evidence of *F*_0_ lowering in English voiced obstruents (Hanson, 2009). In the present study, we observed an increasing downward perturbation after the initial perturbation. The lowering effect reaches around 13 Hz after stop-sonorants at the *F*_0_ elbow. It then gradually declines to 5 Hz after voiced stops and 8 Hz after stop-sonorants compared with nasals at the syllable offset. No such perturbation is found after voiced fricatives. Unlike even the longer-lived upward perturbation, this effect shows no sign of abating for stop-sonorants even at the end of our measurement, which was on average 194 ms from the release of the target consonant. Not only is this consistent with Halle and Stevens (1971) hypothesis that the vocal folds are slackened to maintain voicing during a long oral closure when the transglottal pressure drop is quickly reduced below that of phonation threshold (Berry *et al.*, 1996), but also it is first evidence that the voicing contrast is long lasting.

### C. Anticipatory perturbation by obstruent coda consonants

As shown in Figs. 9(a) and 9(b), there are also two kinds of *F*_0_ perturbations by coda consonants. Right before the closure of an obstruent coda, there is a very brief lowering of *F*_0_, which is small in magnitude. Further back in time, there is a much greater perturbation: *F*_0_ preceding voiceless coda consonants is higher than voiced coda. The raising effect starts to appear in the midpoint of the vowel toward the coda closure but does not reach statistical significance until the very last measurement point (Fig. 18). The *F*_0_ contours in CVCV syllables before the second C and those before CV syllables, however, do not differ from one another. Thus, the anticipatory *F*_0_ perturbation does not apply across syllable boundaries.

The anticipatory *F*_0_ perturbation by coda consonants should be taken with caution, however, because they are potentially biased by difficulties in the alignment of obstruent and nasal contours. First, we marked the offsets of final obstruents at the resumption of voicing, if there was any voice break. The oral release, which often precedes the resumption of voicing, would be earlier when the coda is voiceless than when it is voiced. Second, there are significant differences in syllable duration due to the well-known pre-consonantal voicing effect in English (House and Fairbanks, 1953; House, 1961), which might have affected the phonetic implementation of the base *F*_0_ contours. The average duration of target words is 380 ms with final nasals, 398 ms with final voiced stops, 408 ms with final voiceless stops, 411 ms with final voiced fricatives, and 442 ms with final voiceless fricatives. Since our method of measuring perturbation depends on the alignment of obstruent curves to nasals, errors in the placement of a syllable boundary in the nasal contour would result in misalignment to all corresponding obstruents, which would create gaps between the curves that are not due to actual perturbation but are measured as such. Looking from Figs. 9(a) and 9(b), however, even with adjustments in alignment, *F*_0_ before voiceless consonant would still be higher in both statements and questions. Nevertheless, further studies are necessary to fully resolve this issue.

## V. CONCLUSION

The present study is a further effort to improve the understanding of consonantal perturbation of *F*_0_. Recent studies (Hanson, 2009; Kirby and Ladd, 2016; Kirby *et al.*, 2020) have already shown reduced support for the simple rise-fall dichotomy of *F*_0_ movement after voiced versus voiceless consonants (Hombert *et al.*, 1979) illustrated in Fig. 1. These studies have demonstrated the importance of using *F*_0_ of syllables with sonorant onsets as baseline when assessing the perturbation effect by obstruent consonants. The present study has explored further improvements of methodology by first using the entire syllable as the domain of *F*_0_ alignment and time-normalization rather than the conventional alignment of *F*_0_ contours at vowel voice onset. Furthermore, we tried to improve the precision of *F*_0_ extraction by converting F_0_ from individual vocal cycles without heavy smoothing. With these methods, we were able to observe, for the first time, three distinct kinds of vertical F_0_ perturbations. The first is a large but brief raising effect immediately after most of the consonants, which we interpret as likely due to the vibration of only the outer layer of the vocal folds immediately after the consonant release. The second is a longer-sustained increase in F_0_ both before and after voiceless consonants, which is likely due to an increase in the tension of the vocal folds to inhibit voicing during the voiceless consonant. The third is a sustained downward perturbation after voiced stops and stop-sonorant clusters, which is probably due to the slackening of the vocal folds for the sake of sustaining voicing during the stop closure.

The alignment method used in the present study is based on the assumption that underlying pitch targets associated with a syllable is synchronized with the entire syllable rather than with only the syllable rhyme (Xu and Liu, 2006; Xu, 2020). Based on this assumption, while voice breaks may mask continuous *F*_0_ contours, they do not interrupt the underlying laryngeal movements that produce them. The assessment of the vertical *F*_0_ perturbation by consonants should therefore treat voice breaks as internal to the syllable. The hypothetical nature of the synchronization assumption, however, means that the findings of the present study are also provisional and open to alternative interpretations.

## Footnotes

^1^
Although the same paper also included figures that show *F*_0_ contours in syllables with voiced onset stops are similar to those in syllables with sonorant onset, this figure that gives the impression of a robust dichotomy is the most referred to.

^2^
See supplementary material at https://doi.org/10.1121/10.0004239 for individual plots for all participants.

^3^
In Hanson (2009), some of the initial jumps seem to be captured but others are not.

## APPENDIX A

Table VII shows statistical results of the anticipatory *F*_0_ perturbation in CVC syllables.

**TABLE VII.** Likelihood ratio tests of linear mixed models for the *F*_0_ of vowels preceding target consonants in CVC syllables. Significant effects are indicated in bold.

Fixed effects	Chi-square	df	*p*
CVOICE	2.063	1	0.151
CMANNER	0.063	1	0.802
INTONATION	2.950	1	0.086
TIME	29.714	4	**<0.001**
CVOICE:CMANNER	14.866	3	**0.002**
CVOICE:INTONATION	8.257	2	**0.016**
CVOICE:TIME	72.277	4	**<0.001**
CMANNER:INTONATION	6.044	1	**0.014**
CMANNER:TIME	8.381	4	0.079
INTONATION:TIME	154.21	4	**<0.001**
CVOICE:CMANNER:INTONATION	10.748	1	**0.001**
CVOICE:CMANNER:TIME	17.103	8	**0.029**
CVOICE:INTONATION:TIME	1.701	4	0.791
CMANNER:INTONATION:TIME	34.927	4	<0.001
CVOICE:CMANNER:INTONATION:TIME	2.690	8	0.952

## APPENDIX B

Table VIII shows means of closure duration, *F*_0_ onset, and *F*_0_ jump in CVCV syllables.

**TABLE VIII.** Means (standard deviations) of closure duration (ms), onset *F*_0_ (Hz), and *F*_0_ jump (Hz) across consonant types and sentence type in CVCV syllables.

	Statement			Question		
Consonant type	Closure duration (ms)	Onset *F*_0_ (Hz)	F_0_ jump (Hz)	Closure duration (ms)	Onset *F*_0_ (Hz)	*F*_0_ jump (Hz)
Nasal	69(10)	173(55)	NA	63(13)	187(54)	NA
Voiced stop	35(11)	178(50)	−7(16)	35(9)	170(45)	−6(13)
Voiced fricative	76(17)	170(53)	−7(20)	74(18)	199(64)	8(30)
Voiced consonant (excluding nasal)	55(25)	174(51)	−7(18)	55(24)	185(57)	1(24)
Voiceless stop	108(15)	177(55)	9(20)	98(17)	211(58)	16(27)
Voiceless fricative	124(13)	188(61)	24(21)	112(13)	216(55)	18(24)
Voiceless consonant	116(16)	182(53)	16(22)	105(17)	213(57)	17(25)

## APPENDIX C

See Table IX for statistical results of *F*_0_ jump in CVCV syllables.

**TABLE IX.** Likelihood ratio tests of linear mixed models for the *F*_0_ jump of vowels following target consonants in CVCV syllables. Significant effects are indicated in bold.

Fixed effects	Chi-square	df	*p*
CVOICE	16.870	1	**<0.001**
CMANNER	9.683	1	**0.002**
INTONATION	0.891	1	0.345
CVOICE:CMANNER	0.171	1	0.680
CVOICE:INTONATION	3.316	2	0.191
CMANNER:INTONATION	0.895	2	0.639
CVOICE:CMANNER:INTONATION	11.275	5	**0.046**
